# Drug sensitivity and resistance testing identifies PLK1 inhibitors and gemcitabine as potent drugs for malignant peripheral nerve sheath tumors

**DOI:** 10.1002/1878-0261.12086

**Published:** 2017-07-05

**Authors:** Matthias Kolberg, Jarle Bruun, Astrid Murumägi, John P. Mpindi, Christian H. Bergsland, Maren Høland, Ina A. Eilertsen, Stine A. Danielsen, Olli Kallioniemi, Ragnhild A. Lothe

**Affiliations:** ^1^ Department of Molecular Oncology Institute for Cancer Research the Norwegian Radium Hospital Oslo University Hospital Norway; ^2^ Centre for Cancer Biomedicine University of Oslo Norway; ^3^ Institute for Molecular Medicine Finland FIMM University of Helsinki Finland; ^4^ Science for Life Laboratory Solna Sweden; ^5^ Department of Oncology and Pathology Karolinska Institutet Solna Sweden

**Keywords:** drug screen, MPNST, pharmacology, Schwann cell

## Abstract

Patients with malignant peripheral nerve sheath tumor (MPNST), a rare soft tissue cancer associated with loss of the tumor suppressor neurofibromin (NF1), have poor prognosis and typically respond poorly to adjuvant therapy. We evaluated the effect of 299 clinical and investigational compounds on seven MPNST cell lines, two primary cultures of human Schwann cells, and five normal bone marrow aspirates, to identify potent drugs for MPNST treatment with few side effects. Top hits included Polo‐like kinase 1 (PLK1) inhibitors (volasertib and BI2536) and the fluoronucleoside gemcitabine, which were validated in orthogonal assays measuring viability, cytotoxicity, and apoptosis. DNA copy number, gene expression, and protein expression were determined for the cell lines to assess pharmacogenomic relationships. MPNST cells were more sensitive to BI2536 and gemcitabine compared to a reference set of 94 cancer cell lines. *PLK1*,*RRM1*, and *RRM2* mRNA levels were increased in MPNST compared to benign neurofibroma tissue, and the protein level of PLK1 was increased in the MPNST cell lines compared to normal Schwann cells, indicating an increased dependence on these drug targets in malignant cells. Furthermore, we observed an association between increased mRNA expression of *PLK1*,*RRM1*, and *RRM2* in patient samples and worse disease outcome, suggesting a selective benefit from inhibition of these genes in the most aggressive tumors.

**Abbreviations**
AKTAKT: serine/threonine kinase
BRAF: B‐Raf proto‐oncogene, serine/threonine kinase
CI: confidence interval
CTG: CellTiter‐Glo^®^, viability assay
CTX: CellTox‐Green^®^, cytotoxicity assay
DMEM: Dulbecco's modified Eagle medium
DMSO: dimethyl sulfoxide
DSRT: drug sensitivity and resistance testing
DSS: drug sensitivity score
ERK: mitogen‐activated protein kinase
HR: hazard ratio
HSC: human Schwann cells (two batches, HSC1 and HSC2)
HS‐PSS: MPNST cell line
HS‐Sch‐2: MPNST cell line
IC_50_: half maximal inhibitory concentration
KRAS: V‐Ki‐ras2 Kirsten rat sarcoma viral oncogene homolog
MEK: mitogen‐activated protein kinase kinase
MIM: Mendelian inheritance in Man
MPNST: malignant peripheral nerve sheath tumor
mTOR: mammalian target of rapamycin
NAE: NEDD8‐activating enzyme
NEDD8: neural precursor cell expressed, developmentally down‐regulated 8
NF1: neurofibromatosis type 1 (disease)
NF1: neurofibromin 1 (gene/protein)
PI3K: phosphatidylinositol‐4,5‐bisphosphate 3‐kinase
PLK1: polo‐like kinase 1
QC: quality control
RAF: see BRAF
RAS: see KRAS
RPPA: reverse‐phase protein array
RRM1: ribonucleotide reductase catalytic subunit M1
RRM2: ribonucleotide reductase catalytic subunit M2
S1507‐2: MPNST cell line
S462: MPNST cell line
SCF^βTrCP^: Skp, Cullin, F‐box‐containing complex, E3 ligase
SCGF: Schwann cell growth supplement
SCM: Schwann cell medium
SSMD: strictly standardized mean difference
ST8814: MPNST cell line
STR: short tandem repeats
STS26T: MPNST cell line
TOP2A: topoisomerase (DNA) II alpha
TP53: tumor suppressor protein p53
YST‐1: MPNST cell line

## Introduction

Malignant peripheral nerve sheath tumors (MPNST) are rare and aggressive soft tissue cancers that arise from cells of neuroectodermal origin in the peripheral nervous system. MPNST often strikes young adults and adolescents, and nearly half of all cases are associated with the genetic syndrome neurofibromatosis type 1 (NF1, MIM 162200). The median age for the NF1‐associated cases is around 25 years, while the sporadic cases have a median in the forties (Kolberg *et al*., 2013). The 5‐year overall survival rate for MPNST is less than 50%, and in recent years, the prognosis has been similar for NF1 and non‐NF1 patients (Kolberg *et al*., 2013). There are currently no consensus guidelines for adjuvant treatment with curative intent for MPNST (Bradford and Kim, 2015), and there is a critical need for new treatment options.

Management of MPNST is currently based on general soft tissue sarcoma guidelines and involves surgery and occasionally chemo‐ and radiotherapy (ESMO, 2014). Some relapse control has been reported following radiotherapy (Yang *et al*., 1998); however, radiation itself can be a significant risk factor, especially for patients with NF1 (Sharif *et al*., 2006). The rareness of MPNST and other soft tissue cancers precludes robust clinical trials, and the trials will often include several sarcoma entities with different genetic composition, tumor biology, and hence different drug response. There are 62 interventional trials listed in the US‐based National Institutes of Health (NIH) database that are eligible for patients with MPNST, and 16 of these are currently open or are recruiting patients (Table S1). However, most trials include several different sarcomas, often leaving the numbers of MPNSTs too low, lacking statistical power to conclusively document any benefit for these patients. Only a handful of trials have focused specifically on MPNST with focus on compounds that target the biological processes involved in MPNST development (Table 1). So far, however, none of these trials have compelled changes in the management of this malignancy. Notably, the SARC006 trial, which tested the effect of the TOP2A inhibitors doxorubicin and etoposide in combination with ifosfamide, reported an overall response rate of 33% in the sporadic and 17% in NF1‐associated MPNST, respectively, although both were below the set target of 40% (Widemann *et al*., 2013). The *TOP2A* gene has previously been identified as amplified and upregulated in a large subset of MPNST patient samples (Skotheim *et al*., 2003), which could explain the positive effect of *TOP2A* inhibition in these patients.

**Table 1:** Clinical trials with main focus on MPNST^a^

Trial ID	Intervention	Drug class	Phase	Patient enrollment	Status and results
NCT01661283 (SARC016)	Bevacizumab/everolimus	Cell surface receptor antibody and mTOR inhibitor	II	17 NF1‐associated MPNST, 8 sporadic MPNST	Active, not recruiting Clinical benefit rate (CBR): 12% (3 of 25) (Widemann *et al*., 2016)
NCT00464620 (SARC009)	Dasatinib	Kinase inhibitor (KIT Src)	II	14 MPNST	Active, not recruiting All progressed within 4 months (Schuetze *et al*., 2016)
NCT00304083 (SARC006)	Doxorubicin/ifosfamide followed by etoposide/ifosfamide	Conventional chemotherapy	II	33 NF1‐associated MPNST, 15 sporadic MPNST	Completed Overall response rate 17% in NF1, 33% in sporadic (Widemann *et al*., 2013)
NCT00068367	Erlotinib	Kinase inhibitor	II	24 MPNST; 20 patients were evaluable for response.	Completed One stable disease; 19 no response. Median progression‐free survival: 2 months. Median overall survival: 4 months (Albritton *et al*., 2006)
NCT02008877 (SARC023)	Ganetespib, sirolimus	HSP inhibitor and mTOR inhibitor	I/II	38 MPNST	Active, not recruiting
NCT01418001	Pazopanib in combination with gemcitabine and docetaxel	Kinase inhibitor and conventional chemotherapy	I/II	5 sarcoma	Terminated
NCT00427583	Imatinib mesylate	Kinase inhibitor	II/III	7 MPNST	Terminated All were taken off study; 5 due to progressive disease, one due to toxicity, and one withdrawal (Chugh *et al*., 2009)
NCT02691026	Pembrolizumab	Cell surface receptor antibody	II	18 MPNST	Recruiting
NCT02584647	Pexidartinib (PLX3397), sirolimus	Kinase inhibitor, mTOR inhibitor	I/II	49 MPNST	Recruiting
NCT00837148	Sorafenib, dacarbazine	Kinase inhibitors (BRAF and VEGFR) and conventional chemotherapy	II	12 MPNST evaluated	Completed. All progressed within 12 months. Mean PFS: 1.7 months. Two patients with MPNST had regression or cystification of metastatic disease without a RECIST response (Maki *et al*., 2009)

a Data from clinicaltrials.gov.

Malignant peripheral nerve sheath tumors are highly complex malignancies with multiple copy number alterations (Brekke *et al*., 2010; Lothe *et al*., 1996; Mertens *et al*., 2000) including alterations in several clinically relevant target genes at chromosome arm 17q (Kolberg *et al*., 2015; Skotheim *et al*., 2003; Storlazzi *et al*., 2006). Inactivating mutations in the *NF1* tumor suppressor gene are found in both NF1‐associated and sporadic MPNST (Bottillo *et al*., 2009; Upadhyaya *et al*., 2008). Loss of NF1 activity leads to activation of RAS and consequently contributes to the PI3K/AKT/mTOR and RAF/MEK/ERK signaling in MPNST (Ågesen *et al*., 2005; Berner *et al*., 1999; Brems *et al*., 2009; Danielsen *et al*., 2015; Endo *et al*., 2013; Nielsen *et al*., 1999). Components of this network have been investigated as potential therapeutic targets (Fig. S1). In preclinical MPNST models, some drugs targeting these pathways have shown encouraging results, including the mTOR inhibitors everolimus and AZD8055 (De Raedt *et al*., 2011; Endo *et al*., 2013; Varin *et al*., 2016), and the multikinase Raf inhibitor sorafenib (Ambrosini *et al*., 2008; Castellsague *et al*., 2015), as well as a selection of other drugs that inhibit pathways associated with *NF1*, such as gemcitabine (Schoeler *et al*., 2007), erlotinib (Mahller *et al*., 2007), imatinib (Aoki *et al*., 2007; Patwardhan *et al*., 2014), pexidartinib (Patwardhan *et al*., 2014), and sunitinib (Zietsch *et al*., 2010). However, clinical trials have not confirmed any therapeutic benefit for the limited number of drug candidates identified by a knowledge‐based approach (Table 1). Of note, a recent study suggests the effect of MEK inhibitor selumetinib against inoperable plexiform neurofibromas in children with NF1 (Dombi *et al*., 2016).

As a complement to the knowledge‐based drug discovery approach, we here present a comprehensive high‐throughput approach to identify new therapeutic opportunities for MPNST among a large panel of clinical and investigational drugs. We identify and rank the compounds with the highest effect and specificity for MPNST cells by pharmacological analysis of seven MPNST cell lines using two normal Schwann cell cultures and bone marrow aspirates from healthy donors as controls. Candidate drugs showing the highest selectivity were subjected to validation in independent experiments.

## Material and Methods

### Cell lines, primary cultures, and patient material

The original drug testing assay included two primary cultures of human Schwann cells (HSC) termed HSC1 and HSC2 that were isolated from human spinal nerves (ScienCell, Carlsbad, CA, USA) and four MPNST cell lines, STS26T (Dahlberg *et al*., 1993) and ST8814 (Reynolds *et al*., 1992) (kindly provided by Nancy Ratner, Cincinnati Children's Hospital Medical Center, Cincinnati, OH, USA), and S462 (Frahm *et al*., 2004) and S1507‐2 (Spyra *et al*., 2011) (kindly provided by Lan Kluwe, University Medical Center Hamburg‐Eppendorf, Germany). Later, the three MPNST cell lines HS‐PSS, HS‐Sch‐2 (Sonobe *et al*., 2000), and YST‐1 (Nagashima *et al*., 1990) (Riken BioResource Center, Ibaraki, Japan) were assayed with an extended and updated drug library (see below). STS26T and YST1 were derived from non‐NF1 patients and have been reported to express wild‐type *NF1* (Miller *et al*., 2006; Nagashima *et al*., 1990), and the remaining cell lines are derived from NF1 patients and do not express *NF1*.

All cancer cell lines were maintained in DMEM‐F12 medium supplemented with 10% fetal bovine serum, 2 mm l‐glutamine, 100 units·mL^−1^ penicillin, and 100 μg·mL^−1^ streptomycin (Gibco, Thermo Fisher Scientific, Waltham, MA, USA). The HSC were maintained in Schwann cell medium (SCM, Cat. no. 1701, ScienCell) supplemented with Schwann cell growth supplement (SCGS, Cat. no. 1752, ScienCell) according to the suppliers’ recommendations.

The identity of the cell lines was validated by genotyping of the isolated DNA (Table S2, Appendix S1) according to the protocol of the AmpFLSTR Identifiler PCR Amplification Kit (Life Technologies by Thermo Fisher Scientific). For the cell lines YST‐1, HS‐PSS, HS‐Sch‐2, identical STR profiles were provided by Riken (also available at www.expasy.org/cellosaurus), and for STS26T and ST8814, identical STR profiles were obtained by N. Ratner (personal communication). All the cell lines were tested and found negative for mycoplasma contamination using the MycoAlert detection kit (Lonza Ltd, Basel, Switzerland).

Fresh‐frozen tumor material was available from 30 MPNSTs (17 NF1‐associated and 13 non‐NF1 cases) and eight benign neurofibromas (seven dermal and one plexiform) from Oslo University Hospital, Oslo, Norway, and Skåne University Hospital, Lund Sweden, as previously described (Kolberg *et al*., 2015). Briefly, DNA and RNA were extracted from tissue sections consisting of >90% representative tumor tissue as identified by a reference sarcoma pathologist. Informed consent was obtained from all living patients, and the study was approved by the South‐Eastern Norway Regional Health Authority and the Regional Ethics Committee at Lund University according to national legislation. Bone marrow aspirates were collected from five healthy donors after informed consent using approved study protocols (Helsinki Ethical Committee 239/13/03/00/2010 and 303/13/03/01/2011).

### Drug sensitivity and resistance testing and data analysis

Drug sensitivity and resistance testing (DSRT) was performed as described earlier (Pemovska *et al*., 2013) on all seven MPNST cell lines and two normal HSC cultures. The initial drug library contained 309 compounds, while three MPNST cell lines were screened with 527 compounds (303 overlapping). Reference DSRT data were also available for 299 overlapping drugs for five primary cultures of adult human bone marrow cells derived from healthy donors, and from a reference collection of cell lines from different cancer types, including colorectal (*n *=* *36), ovarian (*n *=* *30), and acute myeloid leukemia (*n *=* *28) (Mpindi *et al*., 2016). Briefly, the compounds were dissolved in 100% dimethyl sulfoxide (DMSO) or water (Table S3) and dispensed on tissue culture‐treated 384‐well plates (Cat. No. 3707, Corning, Tewksbury, MA, USA) using an acoustic liquid handling instrument, Echo 550 (Labcyte Inc., Sunnyvale, CA, USA). The compounds were plated in five concentrations using 10‐fold dilutions covering a 10 000‐fold concentration range (e.g., 1–10 000 nmol·L^−1^). The preprinted plates were kept in pressurized StoragePods (Roylan Developments Ltd., Fetcham, UK) under inert nitrogen gas until needed. Five microlitre of CellTox‐Green (CTX) Cytotoxicity Assay Reagent (Promega, Fitchburg, WI, USA), 1 : 200 dilution in growth media, was added to each 384‐well plate prior to seeding of cells to achieve a final concentration of 1 : 1000. The CTX assay is based on quantification of fluorescence‐labeled DNA released from disrupted cells. Plates were subsequently centrifuged briefly and put on an orbital shaker for 10 min. Twenty microliters of single‐cell suspension (750–1000 cells) was transferred to each well using a Multidrop Combi Reagent Dispenser (Thermo Fisher Scientific). Proliferation rates and growth patterns of the cell lines were evaluated prior to the experiments in order to determine the optimal cell seeding density to assure logarithmic growth throughout the 72‐h incubation period. The plates were incubated in a humidified environment at 37 °C and 5% CO_2_, and after 72 h, cell cytotoxicity and cell viability (assessed using CellTiter‐Glo (CTG) Luminescent Cell Viability Assay, Promega) were measured according to the manufacturer's instructions with a PHERAstar FS microplate reader (BMG Labtech GmbH, Ortenberg, Germany). The CTG assay generates luminescence proportional to the amount of ATP that is extracted from living cells in the culture. The data were normalized to negative control (0.1% DMSO) and positive control wells (containing 100 μm benzethonium chloride, effectively killing all cells). Quality control (QC) metrics, *Z*′, and strictly standardized mean difference (SSMD, i.e., the effect size) for each plate were calculated as described (Mpindi *et al*., 2015; Yadav *et al*., 2014). Drug sensitivity scores (DSS) were calculated for both the CTG assay (DSS_CTG_) and the CTX assay (DSS_CTX_) by fitting of the dose–response curves on the basis of a four‐parameter logistic fit function defined by the top and bottom asymptote, the slope, and the inflection point (IC_50_). In the curve fitting, the bottom asymptote of the curve was fixed to 0% inhibition (=100% viability), whereas the top asymptote was allowed to float above 10% inhibition (i.e., drugs causing < 10% inhibition were considered inactive, DSS≡0), and the slope was allowed to float between 0 and 2.5 (Mpindi *et al*., 2015; Yadav *et al*., 2014). For validation, the orthogonal ApoToxGlo Triplex assay (Promega) was performed according to the manufacturer's instructions, in parallel with the CTG assay (using 15 dilutions from 1 000 nm to 0.0625 nm for each drug). The Triplex assay allows for luminescence measurements of caspase 3/7 activity as a measure of apoptosis levels in addition to viability and cytotoxicity in response to drug treatment. Cells treated with 0.1% DMSO were used as negative controls, 10 μm staurosporin was used as positive control for apoptosis, while 100 μm benzethonium chloride was used as positive control for viability and cytotoxicity.

The average quality control value (*Z*′) for all cell lines in the viability assay was 0.72 ± 0.04 and average SSMD was 14.1 ± 2.4 for each drug plate (Table S4A). The two cell lines S462 and S1507‐2 were re‐tested for technical validation demonstrating high reproducibility of the CTG data [Pearson's correlation *r *=* *0.990 and 0.975, respectively (Fig. S2A and S2B)]. For the two independent normal HSC cultures, the Pearson's correlation was *r *=* *0.982 (Fig. S2C). Overall, there was also a strong correlation in drug response patterns between the MPNST cell lines and the HSC primary cultures (Pearson's correlation *r *=* *0.924) and to a lesser extent between MPNST and bone marrow (Pearson's correlation *r *=* *0.752) (Fig. S2D). For the cytotoxicity assay, three cell lines (S462, YST‐1, and HS‐Sch‐2) failed the quality control. The remaining cell lines had an average *Z*′ of 0.58 ± 0.13 and an average SSMD of −10.5 ± 3.0 (Table S4B).

### Reverse‐phase protein array analyses

Expression of 297 cancer‐related proteins and phosphoproteins was evaluated by reverse‐phase protein array analyses (RPPA) at the MD Anderson RPPA core facility (Houston, TX, USA) in the four MPNST cell lines, S1507‐2, S462, ST8814, and STS26T, and normal HSC1 according to the published protocol (Tibes *et al*., 2006). Later, the MPNST cell lines YST‐1, HS‐PSS, and HS‐Sch‐2, as well as a replicate of the HSC1, were submitted to the same analysis using an updated RPPA version including 306 antibodies, of which 271 were overlapping with the initial 297 antibodies.

### Gene expression analysis

The genome‐wide gene expression levels of the seven MPNST cell lines, HS‐PSS, HS‐Sch‐2, S1507‐2, S462, ST8814, STS26T, and YST‐1, as well as the normal HSC1, were assessed by synthesis of cDNA from isolated RNA and subsequent hybridization to the GeneChip Human Transcriptome Array 2.0 according to the supplier's protocol (Affymetrix, Thermo Fisher Scientific Inc.) (see Appendix S1).

### DNA copy number analyses

DNA from four MPNST cell lines, S1507‐2, S462, ST8814, and STS26T, were individually processed and hybridized on Genome‐Wide Human SNP Array 6.0 from Affymetrix (Thermo Fisher Scientific Inc.) as described in the Affymetrix Cytogenetics Copy Number Assay User Guide (P/N 702607 Rev. 2) (see Appendix S1).

### Mutation analyses

The genes *TP53* (exon 2–11) and *BRAF* (exon 15) were sequenced using DNA extracts of the four MPNST cell lines S1507‐2, S462, ST8814, and STS26T by Sanger sequencing using in‐laboratory‐established protocols (Ahlquist *et al*., 2008; Berg *et al*., 2010) (see Appendix S1).

### Statistical analyses

Association between gene expression and disease‐specific survival was analyzed using Cox proportional hazards regression modeling with Wald test to provide univariate hazard ratios (HR) and confidence intervals (CI) and visualized by Kaplan–Meier plots. Comparison of gene expression differences between MPNST cell lines and other cell lines, and between MPNST patient samples and neurofibromas, was assessed by two‐tailed Student's *t*‐test for independent samples, and correlations between drug screen data from repeated or separate runs were assessed by Pearson's test. Spearman's correlation was used to compare *IC*_50_ from different screening platforms to reduce influence of outliers. All statistical analyses were performed using the spss 21 software (IBM Corporation, Armonk, NY, USA).

## Results

### Identification of MPNST‐specific drugs

**Figure 1:**
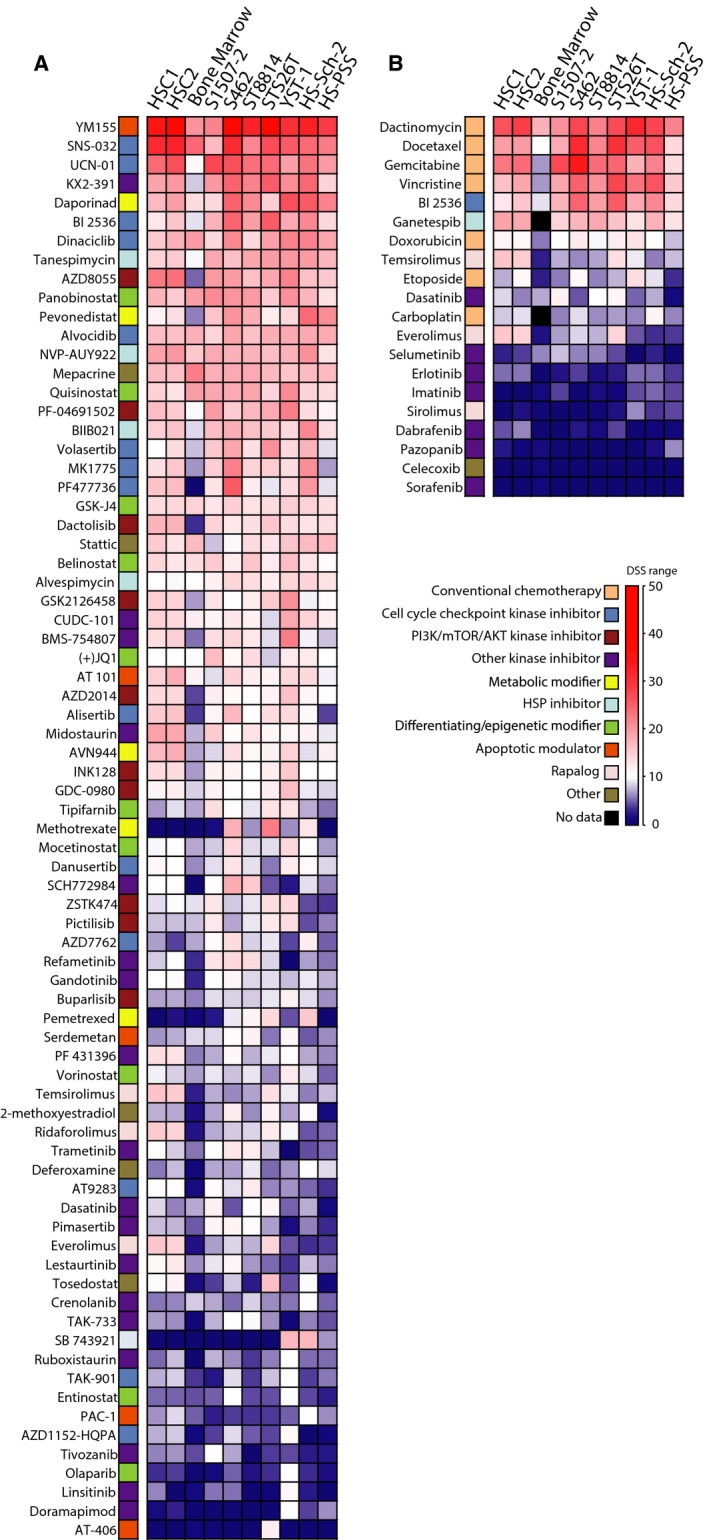
Drug response heatmaps from CellTiter‐Glo (CTG) viability assay for seven MPNST cell lines, two normal human Schwann cell (HSC) cultures, and bone marrow (mean result from five healthy individuals). Drug responses for targeted drugs (excluding chemotherapeutic drugs) with a drug sensitivity score (DSS_CTG_) of 10, or more, in at least one MPNST cell line (A), and chemotherapeutic and other targeted drugs that have been used in clinical treatment of patients with MPNST (B). The same color coding and DSS gradient is used for both heatmaps.

Four MPNST cell lines S1507‐2, S462, ST8814, and STS26T and two HSC primary cultures were subjected to high‐throughput DSRT with 309 emerging and clinical oncology compounds. Three additional MPNST cell lines HS‐PSS, HS‐Sch‐2, and YST‐1 were screened using an updated compound library of 527 compounds (with 303 compounds overlapping between both libraries). Data for 299 of these drugs were also available from normal bone marrow aspirates from healthy donors. As drug sensitivity readout, we used two chemically different assays measuring cell viability (CTG; Fig. 1A; Table S5) and cytotoxicity (CTX; Fig. S3, Table S6).

Twenty of the tested compounds are used in the clinic to treat MPNST (ESMO, 2014), or have been tested in recent clinical trials including patients with MPNST (MPNST trials: Table 1; sarcoma trials: Table S1). Twelve of these showed strong to moderate response in MPNST cells (DSS_CTG _> 5) in our assay, and they also showed differential response in MPNST cells as compared to bone marrow cells (missing data for ganetespib and carboplatin in bone marrow) (Fig. 1B). Only three, docetaxel, vincristine, and BI2536, showed selectively higher response in MPNST cells as compared to HSC (Fig. 1B, Table S5). Strikingly, both mTOR inhibitors temsirolimus and everolimus appear to be more effective in normal HSC than in MPNST cells, while sirolimus did not inhibit any of the cells in our assay at the concentrations used.

**Figure 2:**
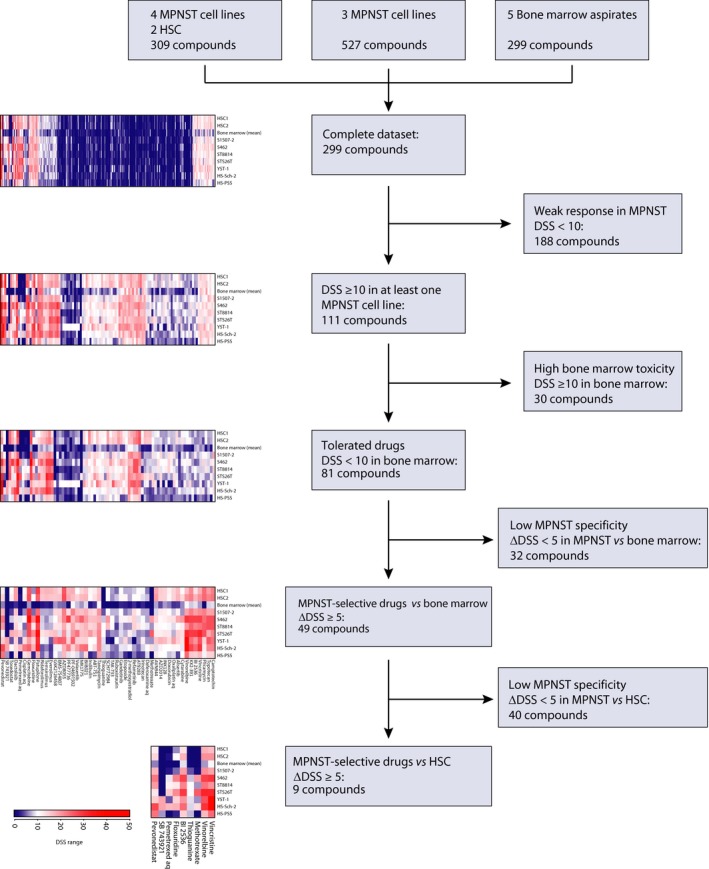
Identification of candidate drugs for MPNST treatment. Filtering steps used to identify drugs with high specificity and selectivity toward MPNST as compared to bone marrow and normal human Schwann cells (HSC) based on drug sensitivity scores from CellTiter‐Glo viability assay (DSS_CTG_).

To systematically identify the most potent drugs, the DSS_CTG_ values were filtered according to MPNST specificity and off‐target toxicity (Fig. 2). Of the 299 drugs tested in all cell types, including bone marrow, 111 had DSS_CTG_ ≥ 10 in at least one MPNST cell line. Eighty‐one of these were well, or moderately, tolerated in bone marrow cells (DSS_CTG_ < 10 in bone marrow), and of these, 49 drugs showed differential sensitivity in MPNST cells with five or more DSS_CTG_ units higher in MPNST cells as compared to normal bone marrow. Nine of these drugs also showed higher selectivity for MPNST cells as compared to HSC (ΔDSS_CTG(MPNST vs. HSC) _≥ 5). These included the polo‐like kinase 1 (PLK1) inhibitor BI2536, three tubulin/kinesin inhibitors (vinorelbine, vincristine, and SB 743921), two nucleoside analogs (floxuridine and thioguanine), two folate analogs (methotrexate and pemetrexed), as well as one proteasome‐ubiquitin inhibitor [NEDD8‐activating enzyme (NAE) inhibitor pevonedistat]. Four of these nine compounds were selected for validation: BI2536 was selected as a targeted kinase inhibitor, floxuridine was selected due to its association with thymidine kinase 1 (TK1), previously identified as a prognostic biomarker (Kolberg *et al*., 2015), while methotrexate and pemetrexed were selected for their differential response in MPNST cell lines. In addition, a second PLK1 inhibitor, volasertib, and a second fluoronucleoside, gemcitabine, were included from the list of 49 compounds with selectivity toward MPNST cells over bone marrow cells. Three of these six compounds, BI2536, volasertib, and gemcitabine, also showed a strong cytotoxic effect (DSS_CTX_ > 10) in the MPNST cells, while methotrexate, pemetrexed, and floxuridine showed limited or no cytotoxicity in the CTX assay (Table S6).

**Figure 3:**
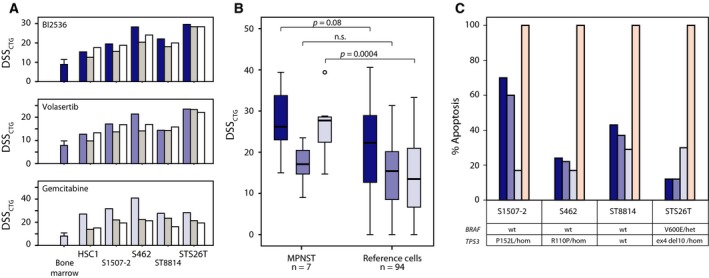
Independent validation and apoptosis assay of MPNST‐specific drugs. Comparison of drug sensitivity scores from initial (blue bars, including average data and standard deviation for the five bone marrow samples) CellTiter‐Glo viability assay (DSS_CTG_), and two subsequent validation rounds [manual (medium gray bars); custom plate (white bars)] (A). DSS_CTG_ obtained for the three drugs BI2536 (dark blue), volasertib (medium blue), and gemcitabine (light blue) from MPNST cell lines in comparison with a reference set of 94 cancer cell lines (colon, ovarian, and leukemia); two‐tailed *P*‐values from independent samples *t*‐test, assuming unequal variance (B). The maximum level of apoptosis measured by a luminescence‐based caspase‐3/7 activation assay, induced by BI2536 (dark blue), volasertib (medium blue), and gemcitabine (light blue), in comparison with staurosporin (100% apoptosis, pink) and 0.1% DMSO (0% apoptosis) (C). The mutation status of *TP53* and *BRAF* in each cell line is shown (het—heterozygous; hom—homozygous).

The validation experiments for BI2536, volasertib, gemcitabine, methotrexate, pemetrexed, and floxuridine were performed using orthogonal assays in four MPNST cell lines (Fig. S4). A good correlation between the initial screen and the validations was observed for the two PLK1 inhibitors, BI2536 and volasertib, as well as for gemcitabine (Fig. 3A). However, the high drug responses for methotrexate, pemetrexed, and floxuridine observed in the initial DSRT were not confirmed (Fig. S4). Interestingly, the DSS_CTG_ values of BI2536 and volasertib appear to be slightly higher for the seven MPNST cell lines as compared to the DSS_CTG_ values of a panel of 94 cell lines from colon and ovarian cancer and leukemia (Fig. 3B), and significantly higher in MPNST cells for gemcitabine. Notably, in the extended drug panel consisting of 527 compounds tested on the three MPNST cell lines HS‐PSS, HS‐Sch‐2, and YST‐1 only, another PLK1 inhibitor, GSK‐461364, showed even higher DSS_CTG_ values for all three cell lines than BI2536 and volasertib (Table S5), while the PLK1 inhibitor TAK‐960 did not inhibit these cells. High DSS values were also observed for the kinase inhibitor rigosertib, which is an inhibitor of both PLK1 and PI3K.

### Cellular responses on specific compounds

The apoptotic response to specific compounds was measured using a photometric caspase 3/7 assay in the four cell lines S1507‐2, S462, ST8814, and STS26T after 72 h of drug exposure normalized to cells treated with staurosporin as positive control. We found that both PLK1 inhibitors, BI2536 and volasertib, induced apoptosis in the *TP53‐*mutant cell lines S1507‐2 and S462, as well as in the *TP53* wild‐type cells ST8814, while the STS26T cell line, which harbors a homozygous 10‐bp deletion in exon 4 of *TP53*, had the lowest level of apoptosis induced by PLK1 inhibition (Fig. 3C). In the presence of gemcitabine, all the four cell lines showed a moderate apoptotic response at 15–30% under our assay conditions (Fig. 3C).

One of the MPNST cell lines, STS26T, had an oncogenic V600E mutation in *BRAF* (Fig. 3C), which is a known marker for benefit of BRAF inhibition in melanoma. We only detected a weak sensitivity against the five tested BRAF inhibitors, RAF265, vemurafenib, regorafenib, dabrafenib, and sorafenib in STS26T, with similar results found for the *BRAF* wild‐type cell lines. Actually, the normal HSCs were moderately more sensitive than all the MPNST cell lines.

### Gene and protein expression of drug targets in MPNST

**Figure 4:**
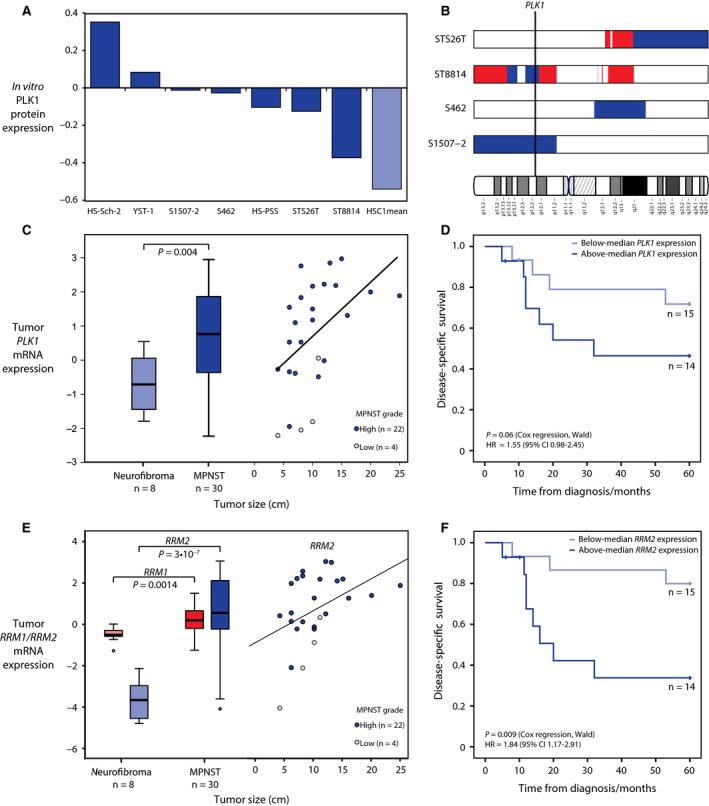
Expression of the drug targets PLK1, RRM1, and RRM2 in MPNST cell lines and prognostic relevance in patients with MPNST. Normalized RPPA protein expression of PLK1 in MPNST cell lines (median = 0 across 271 cancer‐relevant proteins) indicates an increased expression of *PLK1* in seven MPNST cell lines as compared to the mean of duplicate runs of normal Schwann cell HSC1 (A). Regions of genomic gain (red) are not observed in the region on chromosome 16 harboring *PLK1* (16p12.2) in the tested MPNST cell lines, while regions of loss (blue) are observed in the two cell lines S1507‐2 and ST8814 (B). Gene expression of *PLK1* is significantly higher in MPNST tumor samples as compared to benign neurofibromas (C, left panel), and high expression in MPNST is associated with high tumor grade and large tumor size (C, right panel). MPNST patients with high expression of *PLK1* in the tumor had worse outcome than patients with low expression, although not significantly at a 5% level (the *P*‐value and hazard ratio (HR) for *PLK1* expression as a continuous variable in univariate Cox regression analysis for five‐year disease‐specific survival are shown) (D). The gene expression of gemcitabine target *RRM1* and its activator *RRM2* is significantly higher in MPNST tumor samples as compared to benign neurofibromas (E, left panel), and high expression of *RRM2* in MPNST is associated with high tumor grade and large tumor size (E, right panel). MPNST patients with high expression of *RRM2* in the tumor had significantly worse outcome than patients with low expression (*P*‐value and hazard ratio (HR) for gene expression as a continuous variable in univariate Cox regression analysis for 5‐year disease‐specific survival are shown) (F).

The expression of drug targets in the MPNST cell lines and HSC was examined by exon‐level gene expression arrays and protein expression arrays *in vitro*. On the gene expression level, there was little variation in the expression of *PLK1* between MPNST cell lines and HSC (Fig. S5A). On the protein level, however, we found that the expression of PLK1 was higher in the MPNST cell lines as compared to normal HSC (Fig. 4A). Among all the 271 tested proteins on the RPPA array, PLK1 ranked among the top 10 with respect to difference between MPNST and normal cells (Table S7), suggesting that PLK1 is an accessible target in MPNST cells. The increased expression of PLK1 in MPNST as compared to HSC was not associated with gain of gene copy number, as assessed in four MPNST cell lines. Actually, two of the cell lines, S1507‐2 and ST8814, had genomic losses from a chromosomal region covering PLK1 (16p12.2), and for ST8814, this may partly explain the relatively low PLK1 protein level as compared to the other MPNST cell lines (Fig. 4B).

Gene expression data were also available for 30 MPNST patient samples and eight benign neurofibromas (Kolberg *et al*., 2015), and in these patient samples, the gene expression of *PLK1* was significantly upregulated in malignant tumors as compared to benign tumors (*P *=* *0.004, two‐sided independent samples *t*‐test with equal variance; Fig. 4C, left panel). Among the 30 MPNST samples, a high level of *PLK1* expression was associated with large tumor size and high tumor grade (Fig. 4C, right panel). Patients with higher *PLK1* expression also showed worse outcome in univariate analysis, although slightly above the 5% significance level (Fig. 4D). The mechanism of action is more complex for gemcitabine, but one of its direct targets is RRM1 where gemcitabine acts as a suicide substrate (Kolberg *et al*., 2004; Pereira *et al*., 2004). We did not observe any significant difference in *RRM1* or its activator and binding partner *RRM2* in the MPNST cell lines as compared to HSC1 (Fig. S5A); however, these genes were both significantly upregulated in MPNST patient tumor samples as compared to benign neurofibromas (Fig. 4E). The level of *RRM2* was positively correlated with tumor grade and size, and strongly associated with poor patient outcome (Fig. 4E,F). For *RRM1*, there was also an association with poor outcome, although not statistically significant in our patient sample series (Fig. S5E). There was no significant difference in gene expression of the three genes in patient samples from non‐NF1‐ and NF1‐associated MPNSTs (Fig. S5B–D).

## Discussion

There is a need for improved treatment options against MPNST, and to this end, we have systematically tested a comprehensive library of approved and investigational compounds to identify drug candidates that show differential inhibition of MPNST cell growth compared to normal Schwann cells and bone marrow cells. This approach was chosen to identify drugs with low neuro‐ and myelotoxicity, which are common dose‐limiting side effects in the clinical setting. Due to the young age at onset and long life expectancy after curative treatment, avoiding systemic side effects is of particular importance for patients with MPNST. The selection thresholds for the identification of potential new drug candidates were chosen to ensure robust selection of candidate drugs based on true biological differences between MPNST cell lines, normal nerve sheath cells, and bone marrow cells. Other dose‐limiting side effects associated with the drugs, such as gastrointestinal, dermatological, and liver toxicities, were not tractable in our preclinical models.

Recently, several landmark studies have demonstrated how cell lines recapitulate the major molecular phenotypes of cancer and have substantiated their value as preclinical model systems to assess a variety of pharmacogenomic relationships with potential therapeutic impact (Barretina *et al*., 2012; Garnett *et al*., 2012; Greshock *et al*., 2010; Haverty *et al*., 2016). Unfortunately, no MPNST cell lines were included in these studies, highlighting the need for a large systematic screen of available and emerging drugs in a panel of MPNST cell lines to select promising candidates for clinical testing. In our study, we present drug response as DSS values which are derived from the area under the dose–response curves for each drug, and this measure has recently been demonstrated to provide better agreement when comparing results from different laboratories than the inflection points of the dose–response curve (*IC*_50_) (Mpindi *et al*., 2016).

The most promising drug candidates identified here include the PLK1 inhibitors volasertib and BI2536, and the fluoronucleoside gemcitabine. *PLK1* plays an important role in progression of the cell cycle and is known to be overexpressed in many different cancer types, which makes this gene an interesting therapeutic target (Abbou *et al*., 2016; Gjertsen and Schoffski, 2015; Gutteridge *et al*., 2016). In patient biopsies of MPNST, we and others have shown expression changes in various cell cycle‐associated proteins (Ågesen *et al*., 2005; Berner *et al*., 1999; Endo *et al*., 2011; Kourea *et al*., 1999; Nielsen *et al*., 1999). Here, we report that *PLK1* is overexpressed in MPNST compared to benign patient samples; in MPNST cell lines, PLK1 protein expression is higher than in normal HSC. However, the increased expression of PLK1 cannot be explained by DNA copy number aberrations, neither in patient samples (Brekke *et al*., 2010) nor in the cell lines reported here. Furthermore, there was no clear difference in mRNA levels between the mean of the MPNST cells and HSC1 cells. This suggests that the PLK1 is stabilized at the protein level, at least in the MPNST cell lines. A possible mechanism for PLK1 stabilization might be the deregulation of the SCF^βTrCP^/proteasome degradation pathway, which has recently been described as a degradation pathway for PLK1 (Giraldez *et al*., 2017).

Ongoing efforts aim to develop PLK1 inhibitors with improved pharmacokinetic and dynamic profiles, and volasertib is currently the most clinically advanced PLK1 inhibitor (Gjertsen and Schoffski, 2015). BI2536 was among the first PLK1 inhibitors to be tested in the clinic, but the efficacy was limited, partly due to the short terminal half‐life. Nevertheless, the drug seemed to be well tolerated (Schoffski *et al*., 2010). The effect of the PLK1 inhibitor TAK‐960 was recently assessed in a panel of sarcoma cell lines, including two MPNST cell lines (Nair and Schwartz, 2015). Nair and Schwartz found that all tested cell lines were sensitive to TAK‐960 at nanomolar concentrations. The authors also found that inhibition of PLK1 in the *TP53*‐mutant MPNST cell lines, both by small compound inhibition and by siRNA‐mediated gene knockdown, led to the induction of polyploidy, which was in contrast to *TP53* wild‐type or *TP53* ^−/−^ sarcoma cell lines where PLK1 inhibition led to G2 arrest and apoptosis (Nair and Schwartz, 2015). In our study, however, we also observed apoptosis in the two *TP53*‐mutant cell lines S1507‐2 and S462, which carry point mutations P152L and R110P, respectively (Fig. 3C). Apparently, the *TP53* status is not sufficient to explain the relationship between PLK1 inhibition and induction of apoptosis. The available data from other cancer cell lines indicated that MPNST cells have a uniquely high sensitivity toward gemcitabine and PLK1 inhibitors (Fig. 3B), which suggests that the biological processes inhibited by these drugs cannot be easily compensated by other pathways in MPNST cells, at least not within the timeframe of the compound screen. The high sensitivity toward PLK1 inhibitors in MPNST as compared to other cancer cell lines may at least in part be linked to the increased RAS signaling due to the loss of tumor suppressor *NF1*. This is supported by studies of colon cancer models showing increased sensitivity toward PLK1 inhibition in KRAS‐mutated cells (Luo *et al*., 2009; Wang *et al*., 2016).

We have previously reported that enzymes in the nucleotide metabolism, in particular thymidine metabolism, are upregulated in MPNST (Kolberg *et al*., 2015). Several drugs interfering with this pathway were indicated as potential candidates in the drug screen performed here, including the fluoronucleosides gemcitabine and floxuridine, the thiopurines thioguanine and mercaptopurine, as well as the folate antagonists methotrexate and pemetrexed. Of these, we validated the effect of gemcitabine, an inhibitor of RRM1 and *de novo* DNA synthesis (Kolberg *et al*., 2004). Gemcitabine is already approved for many different cancer types, including sarcoma (Ducoulombier *et al*., 2016). Resistance against gemcitabine may partly be mediated by metabolic inactivation of gemcitabine catalyzed by cytidine deaminase (CDA) (Gilbert *et al*., 2006). However, we did not see any differences in *CDA* expression levels in the MPNST cells as compared to HSC.

The gene expression data from patients with MPNST and benign tumors suggest that the drug targets PLK1 and RRM1, as well as the RRM1 activator RRM2, are upregulated in malignant tumors and that the level of aggressiveness, as indicated by patient survival, is directly associated with the gene expression levels, especially for *RRM2* (Fig. 4C–F). A continuous supply of deoxyribonucleotides provided by the RRM1/RRM2 complex is required in rapidly growing and dividing cells, and PLK1 is needed to promote cell cycle progression and to avoid apoptosis. Therefore, inhibition of these factors might be especially effective in the most aggressive tumors. In a clinical setting, *PLK1* and *RRM2* expression may have both prognostic and predictive values, as patients with high expression of these genes are most likely to experience disease progression, and at the same time, those with the highest levels are most likely to respond to PLK1 inhibitors and gemcitabine treatment. Interestingly, a recent study in pancreatic cancer cells showed that PLK1 inhibition enhances the effect of gemcitabine, also in gemcitabine‐resistant cells (Li *et al*., 2016). In view of the current results, a therapeutic combination strategy with gemcitabine and PLK1 inhibitors seems rational also for patients with MPNST.

Novel drug targets for MPNST have also been suggested by others based on preclinical findings (Semenova *et al*., 2017; Teicher *et al*., 2015; Yamashita *et al*., 2014). A recent drug screen of 63 sarcoma cell lines, including the two MPNST cell lines MPNST and ST8814, confirmed the heterogeneous responses among different soft tissue cancers (Teicher *et al*., 2015). None of the highlighted drugs in that study were found to be targeting MPNST cells, with the exception of a moderate inhibition by the PARP1 inhibitor talazoparib, and weak effect of selected aurora kinases (TAK‐901, SCH‐1473759, AS‐703569, and ABT‐348) (Teicher *et al*., 2015). However, in the available raw data, which were recorded after 96‐h drug exposure, both PLK1 inhibitors BI2536 and volasertib, as well as gemcitabine, were among the top‐ranked drugs with respect to low *IC*_50_ values, which are in agreement with our own data (Fig. S6).

In conclusion, we have identified two PLK1 inhibitors, BI2536 and volasertib, and the DNA synthesis inhibitor gemcitabine as highly effective against MPNST cells, while being tolerable to normal HSC cells and bone marrow cells, and we propose these drugs as good candidates for future clinical testing, alone or in combination. The expression levels of target genes for these treatments also carry prognostic value, and we advocate for their potential as prognostic and predictive factors for future clinical trials to be further elucidated.

## Author contributions

MK, JB, OK, and RAL involved in conception and design of the study. MK, JB, AM, CHB, MH, IAE, and SAD acquired data (cell culturing, cell assays, gene expression analysis, DNA copy number analysis, etc). All authors analyzed and interpreted the data. MK and JB drafted the manuscript. All authors reviewed and revised the manuscript. OK and RAL provided administrative, technical, or material support. RAL supervised the study.

## Supporting information


**Fig. S1.** Overview of drugs tested in MPNST patients.

**Fig. S2.** Correlation between CellTiter‐Glo (CTG) viability experiments.

**Fig. S3.** Drug cytotoxicity response profiles of MPNST cell lines and normal HSCs.

**Fig. S4.** Dose–response curves.

**Fig. S5.** Gene expression of drug targets in cell lines and patient tumors, and association to patient survival.

**Fig. S6.** Correlation with public dataset.

**Table S1**. List of drugs in current or previous clinical testing against sarcoma, including MPNST (clinical trials.gov).

**Table S2.** STR profiles of MPNST cell lines*.
**Table S3**. List of tested compounds.
**Table S4.** (A) QC‐scores from viability assay (CTG). (B) QC‐scores from cytotoxicity assay (CTX).
**Table S5.** Cell viability assay (CTG) data.
**Table S6.** Cytotoxicity assay (CTX) data.
**Table S7.** Protein expression data from reverse phase protein lysate microarray (RPPA)^a^.
**Table S8.** Primer sequences.

**Appendix S1.** Supplementary methods.
