# Supplementary figures and images for: Drug sensitivity and resistance testing identifies PLK1 inhibitors and gemcitabine as potent drugs for malignant peripheral nerve sheath tumors
Source: Mol Oncol. 2017 Jul 5;11(9):1156–71. doi: 10.1002/1878-0261.12086 (PMC5579334; doi:10.1002/1878-0261.12086)

## Supplementary Figure S1

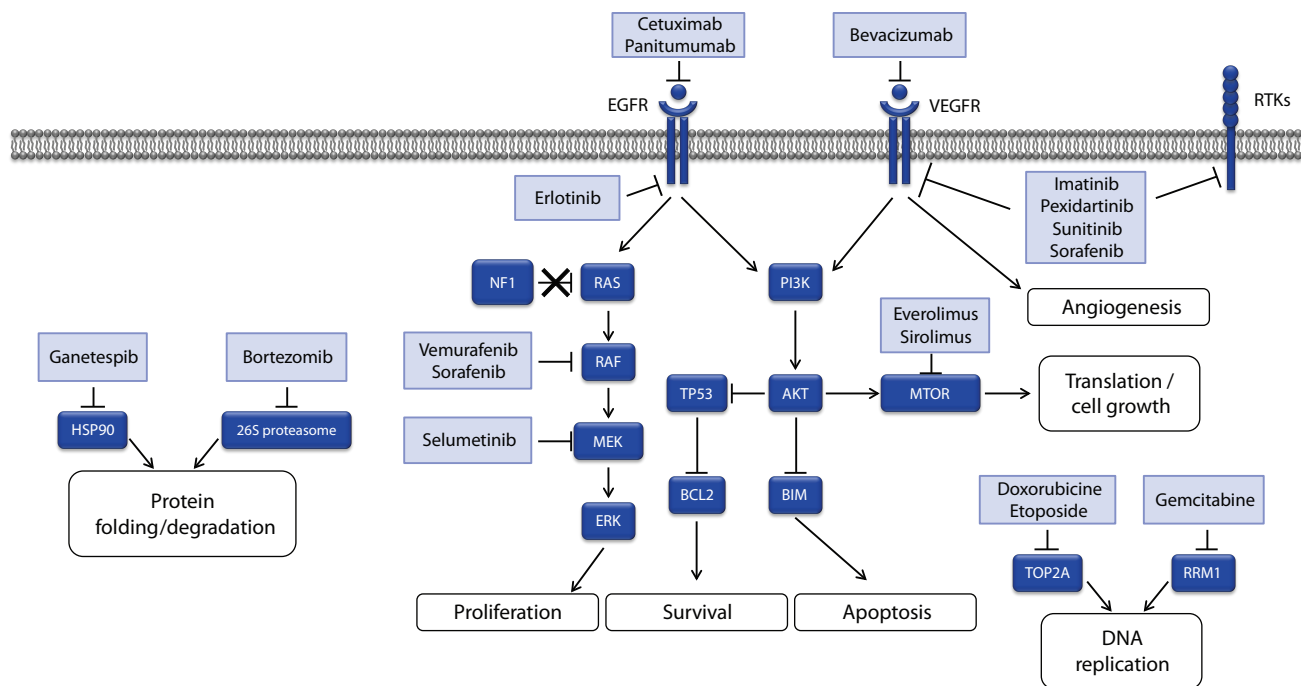

Supplement: Supplementary file 1 — Fig. S1. Overview of drugs tested in MPNST patients. [file MOL2-11-1156-s001.pdf]

Supplementary Figure S2

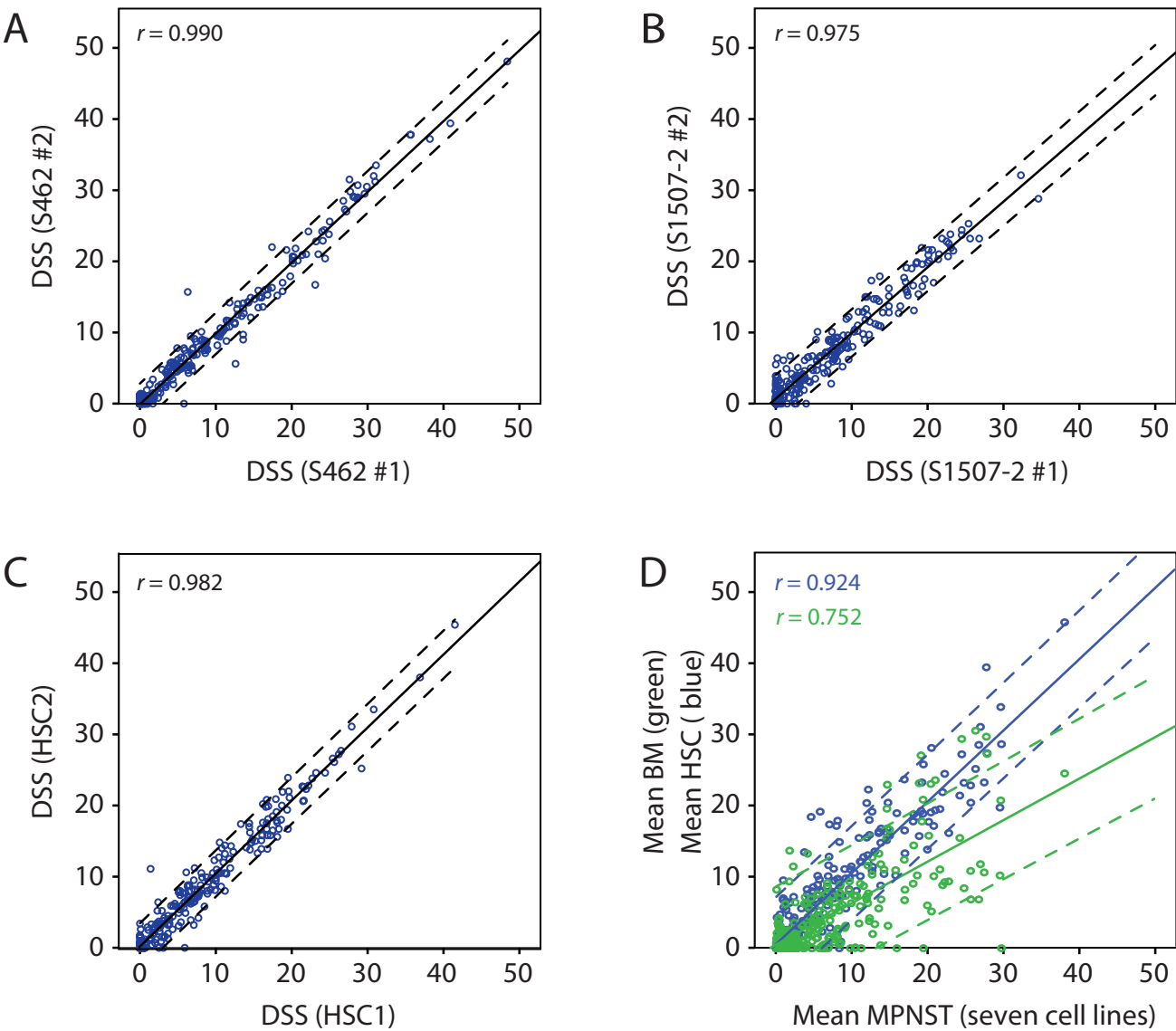

Supplement: Supplementary file 2 — Fig. S2. Correlation between CellTiter‐Glo (CTG) viability experiments. [file MOL2-11-1156-s002.pdf]

Supplementary Figure S3

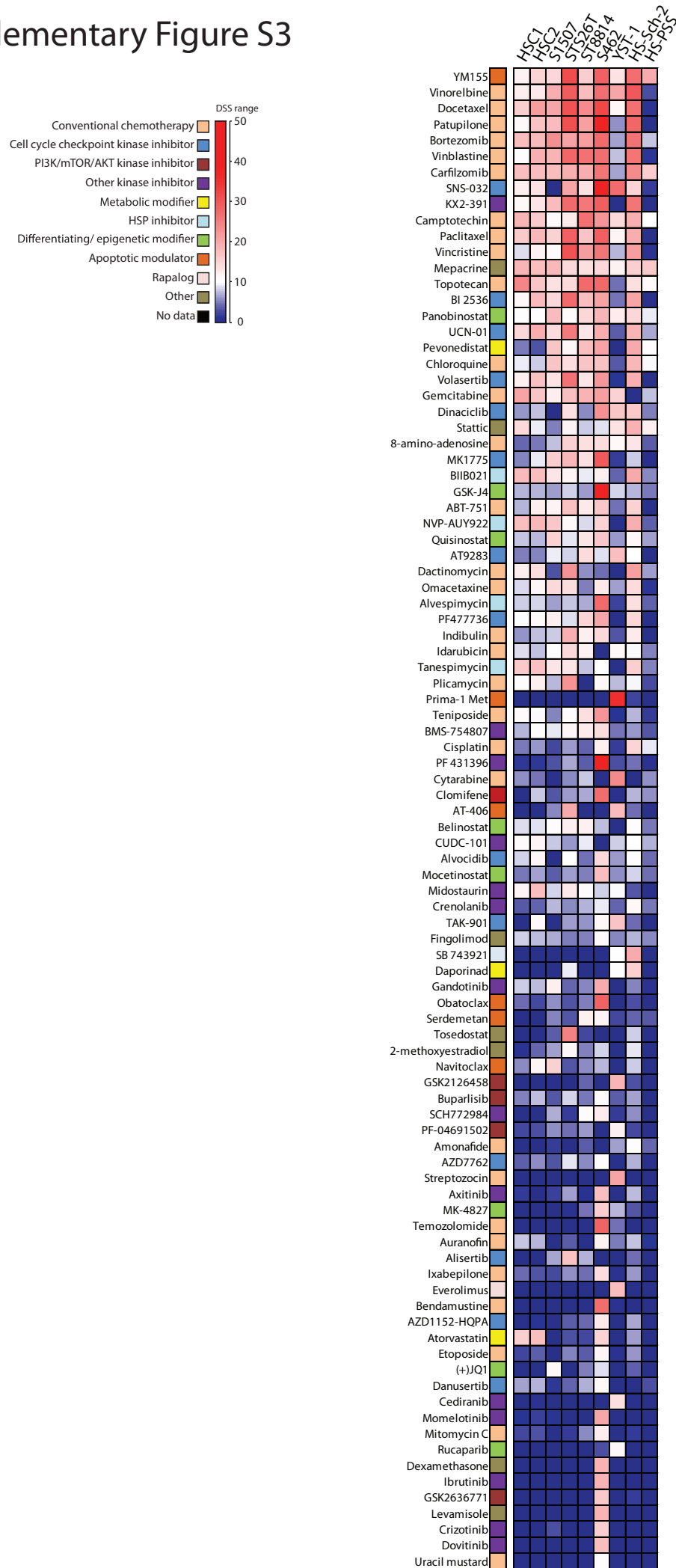

Supplement: Supplementary file 3 — Fig. S3. Drug cytotoxicity response profiles of MPNST cell lines and normal HSCs. [file MOL2-11-1156-s003.pdf]

Supplementary Figure S4

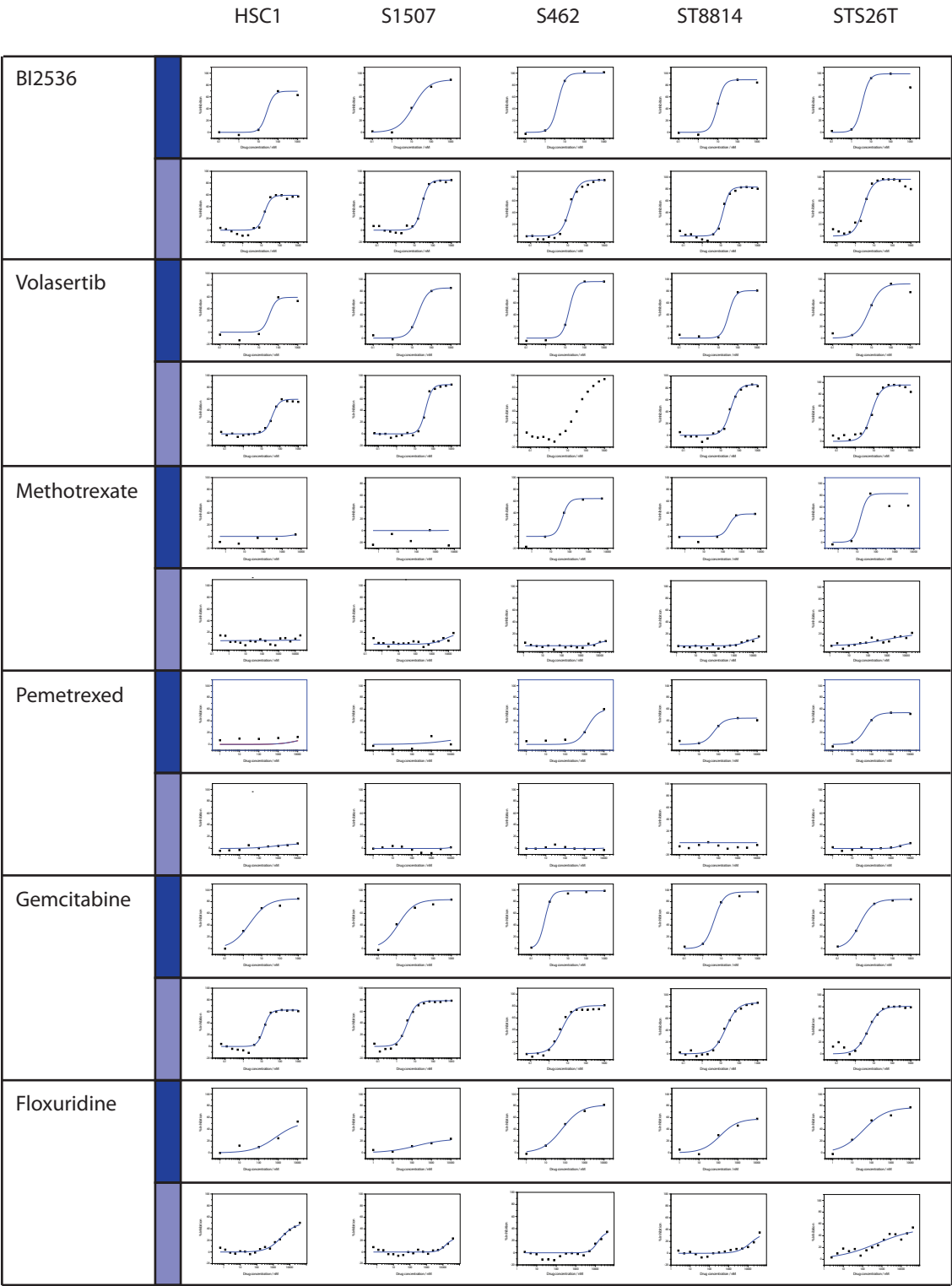

Supplement: Supplementary file 4 — Fig. S4. Dose–response curves. [file MOL2-11-1156-s004.pdf]

# Supplementary Figure S5

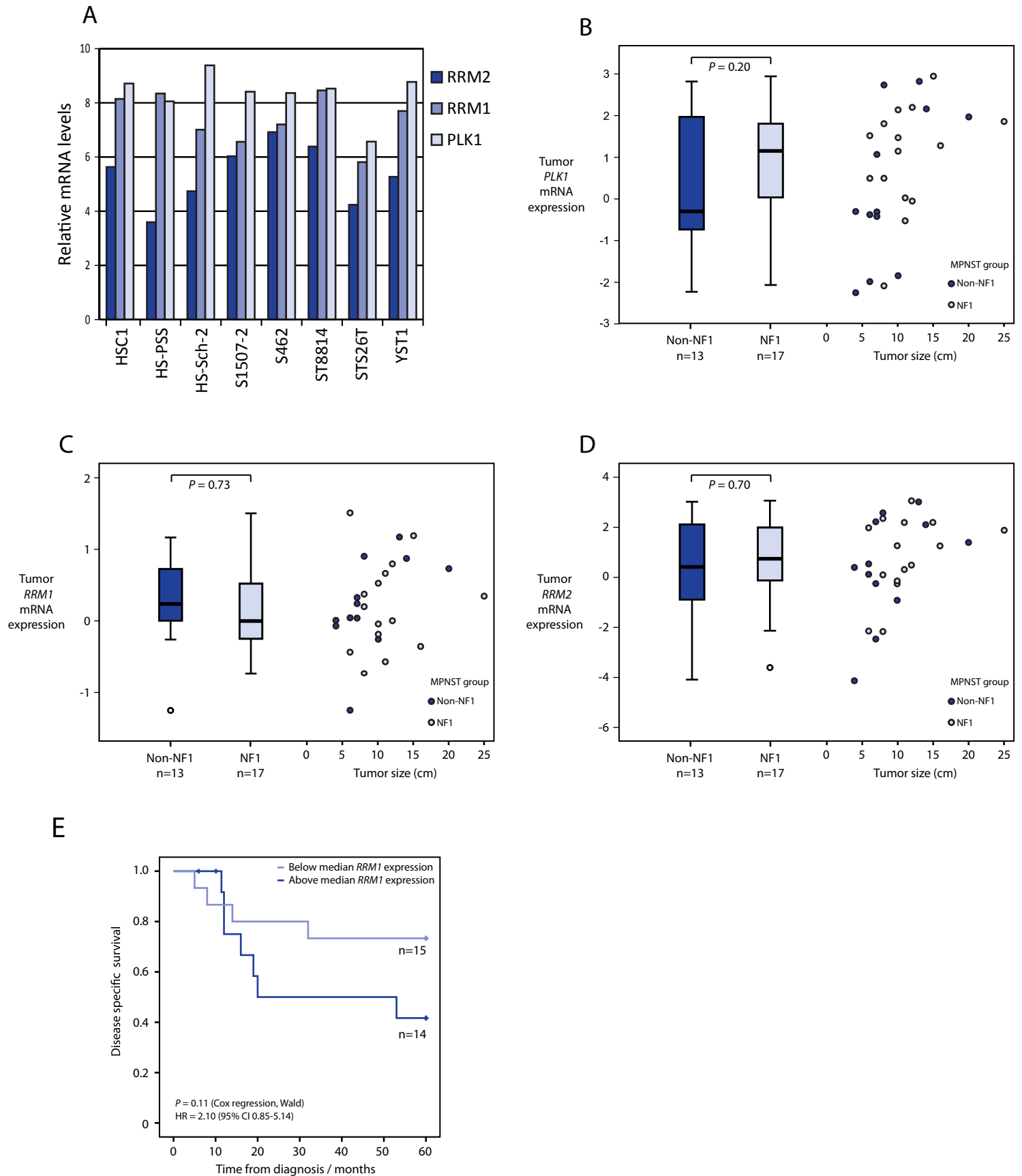

Supplement: Supplementary file 5 — Fig. S5. Gene expression of drug targets in cell lines and patient tumors, and association to patient survival. [file MOL2-11-1156-s005.pdf]

Supplementary Figure S6

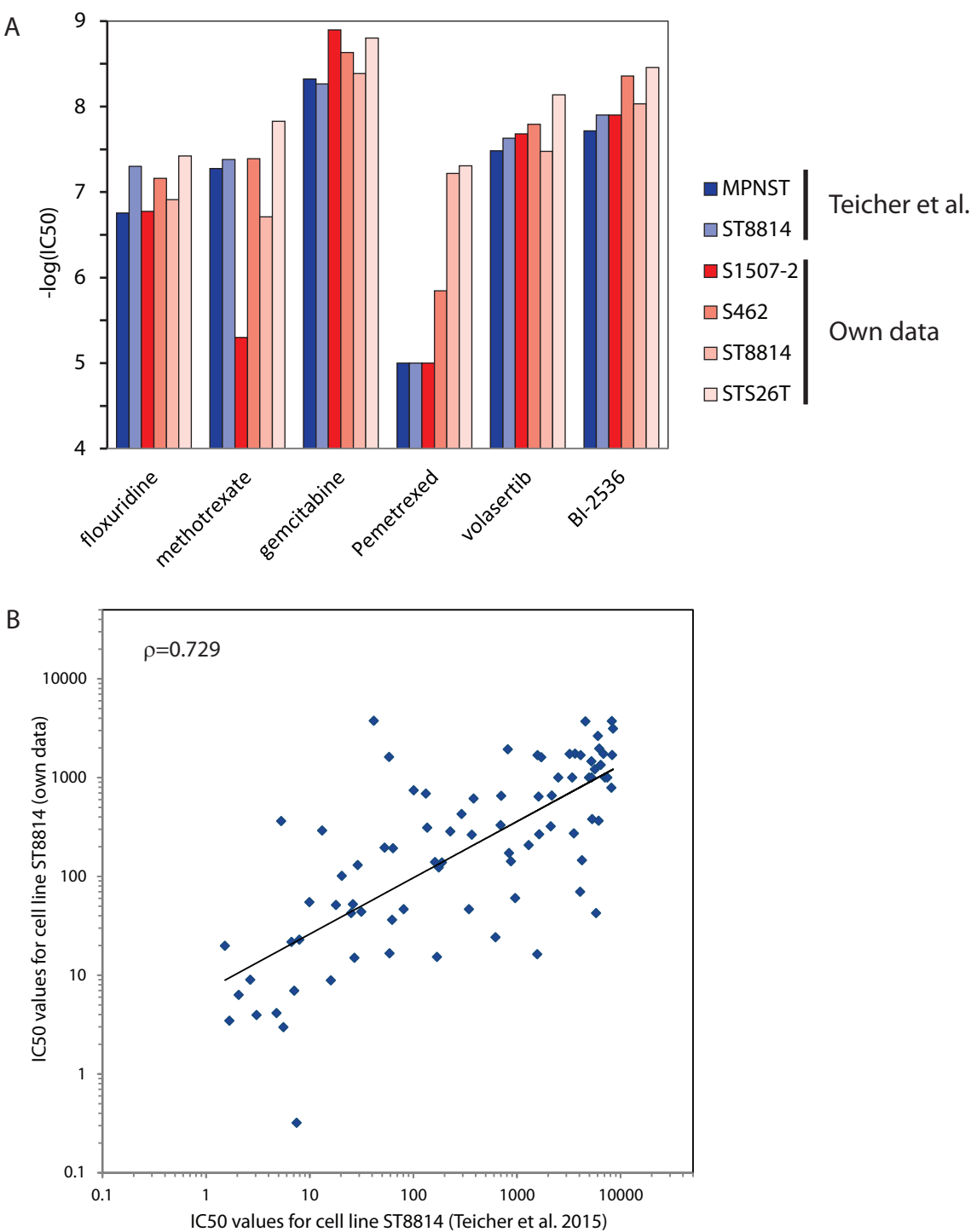

Supplement: Supplementary file 6 — Fig. S6. Correlation with public dataset. [file MOL2-11-1156-s006.pdf]
