# Supplementary material for: Drug sensitivity and resistance testing identifies PLK1 inhibitors and gemcitabine as potent drugs for malignant peripheral nerve sheath tumors
Source: Mol Oncol. 2017 Jul 5;11(9):1156–71. doi: 10.1002/1878-0261.12086 (PMC5579334; doi:10.1002/1878-0261.12086)
Supplement: Supplementary file 7 — Table S1. List of drugs in current or previous clinical testing against sarcoma, including MPNST (clinical trials.gov). [file MOL2-11-1156-s007.docx]

# Supplementary Table S1. List of drugs in current or previous clinical testing against sarcoma, including MPNST (clinical trials.gov)

| Drug name | Drug class | Trial ID: | Patient enrollment | Status |
| --- | --- | --- | --- | --- |
| alisertib | Kinase inhibitor | NCT01653028 | 72 sarcoma | Suspended |
| anlotinib | Kinase inhibitor | NCT01878448 | 200 sarcoma | Active, not recruiting |
| bevacizumab | Cell surface receptor antibody | NCT01661283 | 25 MPNST | Active, not recruiting |
| BI 2536 | Kinase inhibitor | NCT00526149 | 76 patients incl 14 sarcoma | Completed |
| brostallicin* | Conventional chemotherapy | NCT00410462 | 118 patients incl sarcoma | Unknown |
|  |  | NCT00041249 | 64 GIST and sarcoma patients | Completed |
| carboplatin | Conventional chemotherapy | NCT00002898 | 400 pediatric sarcoma | Completed |
| celecoxib | NSAID | NCT00450736 | 3 sarcoma | Completed |
| cixutumumab | Cell surface receptor antibody | NCT00720174 | 30 sarcoma | Completed |
|  |  | NCT01614795 | 46 sarcoma | Active, not recruiting |
| cyclophosphamide | Conventional chemotherapy | NCT00002898 | 400 pediatric sarcoma | Completed |
| cyproheptadine hydrochloride* | antihistamine | NCT01132547 | 30 unspecified cancer patients | Terminated |
| dabrafenib | Kinase inhibitor | NCT02684058 | 30 pediatric high grade glioma, incl MPNST | Not yet recruiting |
| dacarbacine | Conventional chemotherapy | NCT00837148 | 37 sarcoma | Completed |
|  |  | NCT01710176 | 350sarcoma | Recruiting |
| dactinomycin | Conventional chemotherapy | NCT00002898 | 400 pediatric sarcoma | Completed |
| dasatinib | Kinase inhibitor | NCT00464620 | 386 sarcoma | Active, not recruiting |
| dexrazoxane hydrochloride* | Iron chelator | NCT02584309 | 73 sarcoma | Recruiting |
| docetaxel | Conventional chemotherapy | NCT01418001 | 5 sarcoma | Terminated |
|  |  | NCT01710176 | 350 sarcoma | Recruiting |
| doxorubicin hydrochloride | Conventional chemotherapy | NCT00304083 | 74 NF1 + sarcoma | Completed |
|  |  | NCT00346164 | 588sarcoma | Active, not recruiting |
|  |  | NCT02180867 | 340 sarcoma | Recruiting |
|  |  | NCT00720174 | 30 sarcoma | Completed |
|  |  | NCT02584309 | 73 sarcoma | Recruiting |
|  |  | NCT00346125 | 70 sarcoma | Active, not recruiting |
|  |  | NCT01189253 | 133 patients including sarcoma | Terminated |
|  |  | NCT00410462 | 118 patients including sarcoma | Active, not recruiting |
|  |  | NCT00061984 | 455 sarcoma | Completed |
|  |  | NCT00003212 | 780 sarcoma | Completed |
|  |  | NCT00003052 | 340 sarcoma | Completed |
|  |  | NCT00002764 | 340 patients including sarcoma | Completed |
|  |  | NCT00002641 | 350 patients including sarcoma | Completed |
|  |  | NCT00334854 | 250 sarcoma | Recruiting |
|  |  | NCT00949325 | 24 sarcoma | Completed |
| epirubicin hydrochloride* | Conventional chemotherapy | NCT01710176 | 350 sarcoma | Recruiting |
|  |  | NCT00002898 | 400 pediatric sarcoma | Completed |
| erlotinib hydrochloride | Kinase inhibitor | NCT00068367 | 24 sarcoma | Completed |
| etoposide | Conventional chemotherapy | NCT00304083 | 74 NF1 + sarcoma | Completed |
|  |  | NCT01710176 | 350 sarcoma | Recruiting |
|  |  | NCT00003052 | 340 sarcoma | Completed |
|  |  | NCT00002898 | 400 pediatric sarcoma | Completed |
| everolimus | Rapalog | NCT01661283 | 25 MPNST | Active, not recruiting |
| exatecan mesylate* | Conventional chemotherapy | NCT00041236 | 39 sarcoma | Completed |
| ganetespib | HSP inhibitor | NCT02008877 | 38 MPNST | Active, not recruiting |
| gemcitabine hydrochloride | Conventional chemotherapy | NCT01418001 | 5 sarcoma | Terminated |
|  |  | NCT01532687 | 80 sarcoma | Recruiting |
|  |  | NCT01710176 | 350 sarcoma | Recruiting |
| ifosfamide | Conventional chemotherapy | NCT00304083 | 74 NF1 + sarcoma | Completed |
|  |  | NCT00346164 | 588 sarcoma | Active, not recruiting |
|  |  | NCT02180867 | 340 sarcoma | Recruiting |
|  |  | NCT01710176 | 350 sarcoma | Recruiting |
|  |  | NCT00346125 | 70 sarcoma | Active, not recruiting |
|  |  | NCT00061984 | 455 sarcoma | Completed |
|  |  | NCT00053794 | ND | Completed |
|  |  | NCT00030784 | 28 sarcoma | Completed |
|  |  | NCT00003212 | 780 sarcoma | Completed |
|  |  | NCT00003052 | 340 sarcoma | Completed |
|  |  | NCT00002764 | 340 patients including sarcoma | Completed |
|  |  | NCT00002641 | 350 patients including sarcoma | Completed |
|  |  | NCT00334854 | 250 sarcoma | Recruiting |
|  |  | NCT00002898 | 400 pediatric sarcoma | Completed |
| imatinib mesylate | Kinase inhibitor | NCT00427583 | 11 MPNST | Terminated |
|  |  | NCT00154388 | 185 unspecified patients | Completed |
|  |  | NCT00006357 | 91 patients incl sarcoma | Completed |
| lorvotuzumab mertansine | Cell surface receptor antibody | NCT02452554 | 150 sarcoma | Recruiting |
| pazopanib hydrochloride | Kinase inhibitor | NCT02180867 | 340 sarcoma | Recruiting |
|  |  | NCT02601209 | 148 sarcoma | Recruiting |
|  |  | NCT01418001 | 5 sarcoma | Terminated |
|  |  | NCT01532687 | 80 sarcoma | Recruiting |
| pegylated liposomal doxorubicin hydrochloride* | Conventional chemotherapy | NCT00346125 | 70 sarcoma | Active, not recruiting |
|  |  | NCT00030784 | 28 sarcoma | Completed |
| pembrolizumab | Cell surface receptor antibody | NCT02691026 | 18 MPNST | Recruiting |
|  |  | NCT02301039 | 80 sarcoma | Active, not recruiting |
| PLX3397* | Kinase inhibitor | NCT02584647 | 49 MPNST | Recruiting |
| RO4929097* | Gamma-secretase Inhibitor | NCT01154452 | 78 sarcoma | Completed |
| romidepsin | HDAC inhibitor | NCT00112463 | 36 sarcoma | Active, not recruiting |
| sapanisertib | Kinase inhibitor | NCT02601209 | 148 sarcoma | Recruiting |
| sirolimus | Rapalog | NCT02008877 | 38 MPNST | Active, not recruiting |
|  |  | NCT02584647 | 49 MPNST | Recruiting |
| sorafenib | Kinase inhibitor | NCT00837148 | 37 sarcoma | Completed |
|  |  | NCT00245102 | 147 sarcoma | Completed |
| sunitinib | Kinase inhibitor | NCT00474994 | 53 patients including sarcoma | Completed |
| tazemetostat | Epigenetic inhibitor | NCT02601950 | 150 sarcoma | Recruiting |
|  |  | NCT02601937 | 44 sarcoma | Recruiting |
| temozolomide | Conventional chemotherapy | NCT00003718 | 25 patients incl sarcoma | Active, not recruiting |
| temsirolimus | Rapalog | NCT01614795 | 46 sarcoma | Active, not recruiting |
|  |  | NCT00949325 | 24 sarcoma | Completed |
| trabectedin* | Conventional chemotherapy | NCT01710176 | 350 sarcoma | Recruiting |
|  |  | NCT01189253 | 133 sarcoma | Terminated |
|  |  | NCT00003939 | 132 sarcoma | Completed |
| vincristine sulfate | Conventional chemotherapy | NCT00002898 | 400 pediatric sarcoma | Completed |
| vismodegib | Kinase inhibitor | NCT01154452 | 78 sarcoma | Completed |

* not included in our drug library
