# Supplementary material for: Drug sensitivity and resistance testing identifies PLK1 inhibitors and gemcitabine as potent drugs for malignant peripheral nerve sheath tumors
Source: Mol Oncol. 2017 Jul 5;11(9):1156–71. doi: 10.1002/1878-0261.12086 (PMC5579334; doi:10.1002/1878-0261.12086)
Supplement: Supplementary file 8 — Table S2. STR profiles of MPNST cell lines*. Table S3. List of tested compounds. Table S4. (A) QC‐scores from viability assay (CTG). (B) QC‐scores from cytotoxicity assay (CTX). Table S5. Cell viability assay (CTG) data. Table S6. Cytotoxicity assay (CTX) data. Table S7. Protein expression data from reverse phase protein lysate microarray (RPPA)a. Table S8. Primer sequences. [file MOL2-11-1156-s008.docx]

# Supplementary Table S2. STR profiles of MPNST cell lines*

|  | **S1507-2** | **S462** | **STS26T** | **ST8814** | **YST-1** | **HS-Sch-2** | **HS-PSS** |
| --- | --- | --- | --- | --- | --- | --- | --- |
| AMEL_al1 | X | X | X | X | X | X | X |
| AMEL_al2 |  |  |  | Y |  |  | Y |
| CSF1PO_al1 | 11 | 12 | 10 | 9 | 12 | 11 | 10 |
| CSF1PO_al2 | 12 | 13 | 13 | 12 |  | 13 | 12 |
| D13S317_al1 | 9 | 12 | 9 | 12 | 8 | 11 | 8 |
| D13S317_al2 |  |  | 10 |  | 12 |  | 10 |
| D16S539_al1 | 9 | 13 | 12 | 13 | 10 | 11 | 10 |
| D16S539_al2 | 10 |  | 13 |  | 12 | 12 |  |
| D18S51_al1 | 12 | 16 | 17 | 12 | 13 | 13 | 14 |
| D18S51_al2 |  |  | 18 |  |  | 14 | 15 |
| D19S433_al1 | 13 | 14 | 14 | 13 | 13 | 14.2 | 13 |
| D19S433_al2 | 15 |  |  | 14 | 14 |  |  |
| D21S11_al1 | 28 | 29 | 30 | 29 | 29 | 29 | 28 |
| D21S11_al2 |  | 31 | 31 | 32.2 | 31 | 30 | 32 |
| D2S1338_al1 | 18 | 23 | 20 | 17 | 18 | 20 | 19 |
| D2S1338_al2 | 23 |  |  | 23 | 19 |  |  |
| D3S1358_al1 | 17 | 14 | 14 | 15 | 16 | 18 | 16 |
| D3S1358_al2 |  | 17 |  |  | 18 |  |  |
| D5S818_al1 | 11 | 12 | 11 | 12 | 10 | 13 | 9 |
| D5S818_al2 | 12 | 13 | 12 | 13 | 11 |  | 11 |
| D7S820_al1 | 12 | 8 | 8 | 8 | 9 | 8 | 10 |
| D7S820_al2 |  | 10 | 11 |  | 10 | 11 | 12 |
| D8S1179_al1 | 14 | 10 | 13 | 14 | 10 | 14 | 14 |
| D8S1179_al2 |  | 12 | 14 |  | 15 |  | 15 |
| FGA_al1 | 24 | 20 | 22 | 21 | 19 | 23 | 22 |
| FGA_al2 |  |  | 23 |  | 24 | 26 |  |
| TH01_al1 | 9.3 | 8 | 6 | 9 | 7 | 6 | 6 |
| TH01_al2 |  |  | 9.3 |  | 9 | 7 | 9 |
| TPOX_al1 | 11 | 8 | 8 | 11 | 8 | 8 | 8 |
| TPOX_al2 | 12 |  |  | 12 | 11 |  |  |
| vWA_al1 | 15 | 19 | 17 | 16 | 17 | 15 | 14 |
| vWA_al2 |  |  |  |  | 18 | 16 | 15 |

* Obtained from isolated cell DNA using the AmpFLSTR Identifiler PCR Amplification Kit (Life Technologies by Thermo Fischer Scientific, MA, USA)

# Supplementary Table S4A. QC-scores from viability assay (CTG)

|  | Plate 1 | | Plate 2 | | Plate 3 | | Plate 4 | | Plate 5 | | Plate 6 | | Plate 7 | | Plate 8 | | Mean | |
| --- | --- | --- | --- | --- | --- | --- | --- | --- | --- | --- | --- | --- | --- | --- | --- | --- | --- | --- |
|  | Z' | SSMD | Z' | SSMD | Z' | SSMD | Z' | SSMD | Z' | SSMD | Z' | SSMD | Z' | SSMD | Z' | SSMD | Z' | SSMD |
| HSC1 | 0.78 | 19 | 0.8 | 20 | 0.49 | 8 | 0.7 | 13 | 0.72 | 14 |  |  |  |  |  |  | 0.698 | 14.8 |
| HSC2 | 0.81 | 21 | 0.78 | 18 | 0.79 | 19 | 0.75 | 16 | 0.67 | 12 |  |  |  |  |  |  | 0.760 | 17.2 |
| S1507#1 | 0.72 | 13 | 0.73 | 14 | 0.73 | 14 | 0.71 | 14 | 0.73 | 15 |  |  |  |  |  |  | 0.724 | 14 |
| S1507#2 | 0.72 | 14 | 0.69 | 12 | 0.64 | 10 | 0.54 | 8 | 0.6 | 9 |  |  |  |  |  |  | 0.638 | 10.6 |
| S462#1 | 0.75 | 16 | 0.73 | 14 | 0.68 | 12 | 0.67 | 12 | 0.66 | 12 |  |  |  |  |  |  | 0.698 | 13.2 |
| S462#2 | 0.7 | 13 | 0.69 | 12 | 0.7 | 13 | 0.67 | 12 | 0.62 | 10 |  |  |  |  |  |  | 0.676 | 12 |
| ST8814 | 0.76 | 16 | 0.75 | 16 | 0.75 | 15 | 0.7 | 13 | 0.71 | 14 |  |  |  |  |  |  | 0.734 | 14.8 |
| STS26T | 0.82 | 23 | 0.83 | 23 | 0.78 | 18 | 0.71 | 14 | 0.71 | 14 |  |  |  |  |  |  | 0.770 | 18.4 |
| YST-1 | 0.76 | 14 | 0.78 | 15 | 0.8 | 17 | 0.78 | 15 | 0.73 | 12 | 0.82 | 19 | 0.79 | 16 | 0.81 | 18 | 0.780 | 15.8 |
| HS-PSS | 0.73 | 12 | 0.69 | 10 | 0.71 | 11 | 0.76 | 14 | 0.8 | 17 | 0.79 | 15 | 0.7 | 11 | 0.68 | 10 | 0.733 | 12.5 |
| HS-Sch-2 | 0.7 | 11 | 0.69 | 10 | 0.75 | 13 | 0.75 | 13 | 0.64 | 9 | 0.8 | 16 | 0.72 | 11 | 0.71 | 11 | 0.720 | 11.8 |

# Supplementary Table S4B. QC-scores from cytotoxicity assay (CTX)

|  | Plate 1 | | Plate 2 | | | | Plate 3 | | Plate 4 | | | Plate 5 | | | | Plate 6 | | Plate 7 | | | Plate 8 | | Mean | |
| --- | --- | --- | --- | --- | --- | --- | --- | --- | --- | --- | --- | --- | --- | --- | --- | --- | --- | --- | --- | --- | --- | --- | --- | --- |
|  | Z' | SSMD | | Z' | SSMD | Z' | | SSMD | | Z' | SSMD | | Z' | SSMD | Z' | | SSMD | Z' | SSMD | Z' | | SSMD | Z' | SSMD |
| HSC1 | 0.61 | -10 | | 0.55 | -9 | 0.71 | | -13 | | 0.59 | -9 | | 0.57 | -9 |  | |  |  |  |  | |  | 0.606 | -10 |
| HSC2 | 0.5 | -7 | | 0.42 | -7 | 0.69 | | -13 | | 0.11 | -4 | | 0.6 | -9 |  | |  |  |  |  | |  | 0.464 | -8 |
| S1507#1 | -0.29 | -3 | | 0.29 | -6 | 0.04 | | -4 | | 0.06 | -4 | | -2.59 | -1 |  | |  |  |  |  | |  | -0.498 | -3.6 |
| S1507#2 | 0.54 | -9 | | 0.47 | -7 | 0.49 | | -8 | | 0.19 | -5 | | 0.18 | -4 |  | |  |  |  |  | |  | 0.374 | -6.6 |
| S462#1 | 0.4 | -7 | | 0.56 | -9 | 0.03 | | -4 | | 0.03 | -4 | | -1 | -2 |  | |  |  |  |  | |  | 0.004 | -5.2 |
| S462#2 | -0.05 | -3 | | -1.56 | -1 | -0.14 | | -3 | | -0.49 | -2 | | 0.2 | -5 |  | |  |  |  |  | |  | -0.408 | -2.8 |
| ST8814 | 0.76 | -15 | | 0.78 | -17 | 0.74 | | -14 | | 0.84 | -22 | | 0.51 | -7 |  | |  |  |  |  | |  | 0.726 | -15 |
| STS26T | 0.72 | -13 | | 0.56 | -9 | 0.61 | | -10 | | 0.55 | -9 | | 0.71 | -13 |  | |  |  |  |  | |  | 0.630 | -10.8 |
| YST-1 | -0.34 | -3 | | -0.27 | -3 | 0.2 | | -5 | | -0.15 | -3 | | 0.35 | -6 | 0.1 | | -4 | -0.55 | -3 | -0.55 | | -2 | -0.150 | -3.6 |
| HS-PSS | 0.74 | -15 | | 0.74 | -15 | 0.73 | | -15 | | 0.33 | -5 | | 0.68 | -13 | 0.65 | | -11 | 0.76 | -16 | 0.61 | | -9 | 0.655 | -12.3 |
| HS-Sch-2 | -0.47 | -3 | | 0.46 | -7 | 0.21 | | -5 | | 0.1 | -4 | | 0.31 | -6 | 0.43 | | -7 | 0.11 | -5 | 0.42 | | -7 | 0.196 | -5.5 |

# Supplementary Table S7. Protein expression data from reverse phase protein lysate microarray (RPPA)^a^

| Gene name^b^ | S462 | HS-SCH2 | ST8814 | S1507-2 | STS26T | HS-PSS | YST-1 | HSC1#1 | HSC1#2 | FC^c^ | *d*-score^d^ |
| --- | --- | --- | --- | --- | --- | --- | --- | --- | --- | --- | --- |
| HES1 | 0.2 | 0.8 | 0.9 | 0.6 | 0.0 | -0.7 | -0.2 | -0.4 | -1.1 | 2.0 | 1.9 |
| PPIF | -0.7 | 0.6 | -0.9 | -0.6 | 0.0 | -0.4 | -1.0 | -1.2 | -1.5 | 1.9 | 2.2 |
| CDK1 | 0.7 | 0.1 | 0.6 | 0.2 | 0.4 | 0.4 | 0.1 | -0.7 | -0.3 | 1.8 | 3.0 |
| MTCO2P1 | 0.0 |  | -0.5 | -0.6 | -0.6 |  |  | -1.2 |  | 1.8 | 2.8 |
| SOX2 | 0.7 | 0.2 | 1.1 | -0.1 | 0.8 | 1.0 | 1.3 | -0.1 | 0.0 | 1.7 | 2.0 |
| RPS6KA1 | -0.4 | -0.2 | -0.9 | -0.1 | -0.2 | 0.0 | 0.1 | -1.0 | -0.8 | 1.6 | 2.4 |
| MSH2 | 0.1 |  | 0.1 | -0.1 | 0.1 |  |  | -0.5 |  | 1.5 | 7.0 |
| GLUD | 0.0 | -0.2 | 0.2 | 0.3 | -0.1 | 0.0 | 0.0 | -0.5 | -0.5 | 1.5 | 4.6 |
| TFAM | 0.3 | 0.2 | -1.0 | -0.7 | -0.3 | -0.2 | -0.3 | -0.8 | -0.9 | 1.4 | 1.6 |
| **PLK1** | 0.0 | 0.4 | -0.4 | 0.0 | -0.1 | -0.1 | 0.1 | -0.8 | -0.3 | 1.4 | 1.8 |
| SOD1 |  | 0.0 |  |  |  | 0.3 | 0.3 |  | -0.3 | 1.4 | 3.9 |
| CD276 | 0.6 |  | 0.5 | 0.6 | 0.6 |  |  | 1.1 |  | 0.7 | -15.0 |
| CD29 | 0.1 | -0.7 | 0.0 | 0.0 | 0.3 | 0.1 | -0.5 | 0.6 | 0.4 | 0.7 | -2.2 |
| RPS6 | 0.1 | -0.2 | -0.6 | -0.4 | -0.3 | 0.0 | -0.4 | 0.3 | 0.5 | 0.7 | -3.0 |
| MAP1LC3A | 0.6 | 0.9 | 0.7 | 1.0 | 0.3 | 0.8 | 0.5 | 1.5 | 1.2 | 0.6 | -3.2 |
| MYH11 | -0.2 | 0.0 | 0.0 | 0.4 | 0.7 | 0.0 | -0.1 | 1.3 | 0.5 | 0.6 | -1.7 |
| MYH9 pS1943 | -0.1 | -0.5 | 0.2 | 0.5 | 1.0 | 0.6 | 0.1 | 1.5 | 2.0 | 0.4 | -3.4 |
| RPS6 pS240 | -0.9 | -1.6 | 1.6 | 1.3 | 0.9 | 1.4 | 0.0 | 1.9 | 1.9 | 0.4 | -1.7 |
| NR2F2 | -0.2 |  | 0.0 | 0.2 | -0.7 |  |  | 1.3 |  | 0.3 | -4.0 |
| RPS6 pS235 S236 | -0.7 | -1.9 | 1.4 | 1.3 | 1.5 | 2.2 | -0.3 | 2.3 | 2.6 | 0.3 | -1.8 |
| FN1 | 0.3 | 0.4 | 1.5 | 0.9 | 0.9 | 0.6 | 0.4 | 2.6 | 2.7 | 0.3 | -6.4 |
| GJA1 | 3.3 | 0.3 | 2.9 | 3.7 | -0.1 | 2.5 | 1.7 | 4.9 | 4.3 | 0.2 | -2.4 |
| PDGFR | -0.4 | 2.4 | 2.8 | 3.1 | 2.4 | 0.1 | -0.3 | 3.8 | 6.3 | 0.1 | -2.1 |

^a^Only proteins with fold change (FC) or 1/FC ≥ 1.4 and |*d*-score|≥1.5 between MPNST and HSC are shown from a panel of 357 selected antibodies. The colors indicate the highest (red) and lowest (blue) value in each row.

^b^Phospho-specific antibodies are indicated with phosphorylation site

^c^FC – expression fold change between the seven MPNST cells and the two repeats of normal HSC1

^d^*d*-score = $\frac{\mu_{MPNST}-\mu_{HSC}}{\sqrt{1/2\left( \sigma_{MPNST}^{2}+\sigma_{HSC}^{2} \right)}}$ where *µ* is the mean expression for each cell type and *σ* the corresponding standard deviation

# Supplementary Table S8. Primer sequences

| Gene | Fragment | Sense forward | Antisense reverse |
| --- | --- | --- | --- |
| *BRAF* | | | |
|  | Exon 15 | 5’-TCATAATGCTTGCTGTGATAGGA-3’ | 5’-GGCCAAAAATTTAATCAGTGGA-3’ |
| *TP53* | | | |
|  | Exon 2-4 | 5’-M13F_tcagacactggcatggtgtt-3’ | 5’-M13R_gccaaagggtgaagaggaat-3’ |
|  | Exon 4 s | 5’-gacctggtcctctgactgctctt |  |
|  | Exon 5-6 | 5’-M13F_cacttgtgccctgactttca-3’ | 5’-M13R_cttaacccctcctcccagag-3’ |
|  | Exon 7-9 | 5’-M13F_cctgcttgccacaggtct-3’ | 5’-M13R_aagaaaacggcattttgagtg-3’ |
|  | Exon 8-9 s | 5’-ttgggagtagatggagcct |  |
|  | Exon 10 | 5’-M13F_tgcatgttgcttttgtaccg-3’ | 5’-M13R_gaaggcaggatgagaatgga-3’ |
|  | Exon 11 | 5’-M13F_aaagcattggtcagggaaaa-3’ | 5’-M13R_gcaagcaagggttcaaagac-3’ |

s: sequencing primer

M13-primers:

M13F: TGTAAAACGACGGCCAGT

M13R: CAGGAAACAGCTATGACC
