# Supplementary material for: Drug sensitivity and resistance testing identifies PLK1 inhibitors and gemcitabine as potent drugs for malignant peripheral nerve sheath tumors
Source: Mol Oncol. 2017 Jul 5;11(9):1156–71. doi: 10.1002/1878-0261.12086 (PMC5579334; doi:10.1002/1878-0261.12086)
Supplement: Supplementary file 12 — Appendix S1. Supplementary methods. [file MOL2-11-1156-s012.docx]

# Supplementary Methods

**DNA and RNA extraction**

DNA and RNA from the cell lines were isolated using the Allprep DNA/RNA/miRNA Universal Kit as recommended by the manufacturer (QIAGEN, Hilden, Germany). Quantity and quality measurements were carried out using UV spectrometry (NanoDrop ND-1000, Thermo Fisher Scientific, Waltham, MA, USA) and Agilent 2100 Bioanalyzer (Agilent Technologies, Santa Clara, CA, USA). All RNA samples had an RNA integrity number (RIN) of 10.

**Mutation analyses**

DNA was amplified using the Qiagen 2x Multiplex PCR-kit (Qiagen). The primers are listed in Supplementary Table S7. *TP53* exon 10 and exon 11 were amplified simultaneously. When necessary, samples were run in singleplex PCR to obtain evaluable results. *TP53* exon 2-4, exons 5-6, and exons 7-9, were amplified in singleplex using HotStar Taq polymerase (Qiagen). The PCR products were purified enzymatically by illustra ExoProStar 1-Step (GE Healthcare Bio-Sciences, Pittsburgh, PA, USA) prior to a sequencing reaction using the BigDye Terminator v.1.1 Cycle Sequencing Kit (Applied Biosystems, Thermo Fischer Waltham, MA USA) for incorporation of dye labeled ddNTPs. Further, the sequencing reaction was purified with Big Dye Xterminator (Applied Biosystems) before sequencing on ABI 3730 DNA Sequencer (Applied Biosystems). The results were processed using the SeqScape® software, version 2.5 (Applied Biosystems). All results were scored individually by two persons, and positive results were verified by a second, independent round of PCR and sequencing.

**DNA copy number analyses**

Raw probe intensity data from scanned images of the arrays were stored in cell intensity (CEL) files by the Affymetrix Gene Chip Command Console software (version 1.0), and quality control of the individual CEL files was performed using the Affymetrix Genotyping Console software (version 4.1.4.840). All samples had a CEL data quality above the recommended threshold (Contrast QC > 0.4). For copy number analysis the CEL files were preprocessed according to the PennCNV protocol ([Wang et al., 2007](#_ENREF_3)) adapted for Affymetrix genotyping arrays. HapMap samples previously analyzed on the SNP Array 6.0 (n = 270 individuals from four populations) ([McCarroll et al., 2008](#_ENREF_1)) were used as reference for quantile normalization and calculation of Log R Ratio (LRR) and B Allele Frequence (BAF). The LRR values from the four cell lines were subjected to winsorization and single-sample segmentation by the PCF algorithm implemented in the Bioconductor package copynumber (version 1.12.0) ([Nilsen et al., 2012](#_ENREF_2)) (the penalty parameter gamma was set to 100 and the minimum number of probes per segment, k_min_, was set to 5). Copy number estimates >0.15 was called as copy number gain while estimates <-0.15 was called as loss. Plots were generated using the copynumber package.

**Gene expression analyses**

100 ng total cell line RNA was used as input for cDNA synthesis, followed by amplification and DNA sense strand labeling according to the GeneChip Whole Transcript (WT) PLUS Reagent Kit Manual (Affymetrix, Santa Clara, CA, USA). Each sample was hybridized to Affymetrix GeneChip® Human Transcriptome 2.0 arrays for 16 hours and washed, stained and scanned as recommended in the manual. For each sample, a CEL file storing intensity measures was generated by the Affymetrix GeneChip Command Console software (version 4.0.0). The CEL files intensities were adjusted by cytosine count correction and signal space transformation before being further processed through background correction, quantile normalization and summarization at the gene level by robust multiarray average using the Affymetrix Expression Console Software (v1.4.1, HTA-2_0.r3 library files).

**References**

McCarroll, S.A., Kuruvilla, F.G., Korn, J.M., Cawley, S., Nemesh, J., Wysoker, A., Shapero, M.H., de Bakker, P.I., Maller, J.B., Kirby, A., Elliott, A.L., Parkin, M., Hubbell, E., Webster, T., Mei, R., Veitch, J., Collins, P.J., Handsaker, R., Lincoln, S., Nizzari, M., Blume, J., Jones, K.W., Rava, R., Daly, M.J., Gabriel, S.B., Altshuler, D., 2008. Integrated detection and population-genetic analysis of SNPs and copy number variation. Nat Genet 40, 1166-1174.

Nilsen, G., Liestol, K., Van, L.P., Moen Vollan, H.K., Eide, M.B., Rueda, O.M., Chin, S.F., Russell, R., Baumbusch, L.O., Caldas, C., Borresen-Dale, A.L., Lingjaerde, O.C., 2012. Copynumber: Efficient algorithms for single- and multi-track copy number segmentation. BMC.Genomics 13, 591.

Wang, K., Li, M., Hadley, D., Liu, R., Glessner, J., Grant, S.F., Hakonarson, H., Bucan, M., 2007. PennCNV: an integrated hidden Markov model designed for high-resolution copy number variation detection in whole-genome SNP genotyping data. Genome Res 17, 1665-1674.
