# Supplementary material for: Drug sensitivity and resistance testing identifies PLK1 inhibitors and gemcitabine as potent drugs for malignant peripheral nerve sheath tumors
Source: Mol Oncol. 2017 Jul 5;11(9):1156–71. doi: 10.1002/1878-0261.12086 (PMC5579334; doi:10.1002/1878-0261.12086)
Supplement: Supplementary file 13 [file MOL2-11-1156-s013.docx]

Fig. S1. Overview of drugs tested in MPNST patients.

Fig. S2. Correlation between CellTiter-Glo (CTG) viability

experiments.

Fig. S3. Drug cytotoxicity response profiles of MPNST

cell lines and normal HSCs.

Fig. S4. Dose–response curves.

Fig. S5. Gene expression of drug targets in cell lines

and patient tumors, and association to patient

survival.

Fig. S6. Correlation with public dataset.

Table S1. List of drugs in current or previous clinical

testing against sarcoma, including MPNST (clinical trials.

gov).

Table S2. STR profiles of MPNST cell lines*.

Table S4. (A) QC-scores from viability assay (CTG).

(B) QC-scores from cytotoxicity assay (CTX).

Table S7. Protein expression data from reverse phase

protein lysate microarray (RPPA)a.

Table S8. Primer sequences.

Table S3. List of tested compounds.

Table S5. Cell viability assay (CTG) data.

Table S6. Cytotoxicity assay (CTX) data.

Appendix S1. Supplementary methods.
